# AMPK is dispensable for physiological podocyte and glomerular functions but prevents glomerular fibrosis in experimental diabetes

**DOI:** 10.1038/s41420-026-03078-y

**Published:** 2026-03-28

**Authors:** Swayam Prakash Srivastava, Olivia Kopasz-Gemmen, Abhiram Kunamneni, Aaron Thurman, Shota Yoshida, Eden Ozkan, Vinamra Swaroop, Rahul Nanwani, Ajan Arora, Arya Joshi, Om Khuperkar, Mariam Hamed, Mihir Suresh Bharadwaj, Niloy Islam, Adesh Urval, Junying Wang, Sungki Hong, Keizo Kanasaki, Daisuke Koya, Ken Inoki

**Affiliations:** 1 Life Sciences Institute, University of Michigan, Ann Arbor, MI USA; 2 Department of Diabetology and Endocrinology, Kanazawa Medical University, Uchinada, Japan; 3 Department of Molecular and Integrative Physiology, University of Michigan Medical School, Ann Arbor, MI USA; 4 Department of Internal Medicine, University of Michigan Medical School, Ann Arbor, MI USA

**Keywords:** Renal fibrosis, Nephrosclerosis

## Abstract

AMP-activated protein kinase (AMPK) has been postulated to be crucial in regulating various renal physiology and pathophysiology processes, including energy metabolism, ion and water transport, inflammation, and hypertrophy. However, the specific roles of AMPK in podocytes, cells critical for maintaining glomerular filtration, have not been fully explored using genetic model animals. In this study, we generated mice lacking both AMPK α1 and α2 catalytic subunits in glomerular podocytes (pmut). Our findings revealed that, surprisingly, AMPK is dispensable for normal podocyte function. These knockout mice could live as long as their wild-type littermates without showing any pathological alterations in their glomeruli or glomerular function at two years of age. However, under diabetic conditions, the diabetic pmut mice exhibited increased lipid and collagen accumulation and an elevated expression of mesenchymal proteins in their glomeruli. They also showed more significant albuminuria compared to control diabetic mice. Under high-glucose culture conditions, glomeruli isolated from pmut mice showed reduced expression of mitochondrial genes (e.g., Ndufv2) and increased leakage of mitochondrial components. Additionally, there was increased expression of genes associated with nucleotide-sensing and pro-inflammatory pathways (including mb21d2, IL-1β, and NF-κB). These observations suggest that while AMPK is not necessary for podocyte function in healthy kidneys, it is crucial for preventing glomerular fibrosis resulting from lipotoxicity and inflammation under diabetic conditions.

## Introduction

AMP-activated protein kinase (AMPK) is an evolutionarily conserved serine/threonine protein kinase, of which kinase activity is stimulated by various cellular stresses. Upon activation, AMPK stimulates cellular catabolic processes, including beta-oxidation, glycolysis, and autophagy, to restore cellular energy levels. AMPK forms a hetero-trimeric protein complex in which the α subunits contain a kinase domain, while β (β1 and β2) and γ (γ1-3) subunits function as scaffolds and regulatory components in the complex [1, 2]. The mammalian AMPK α subunit has two isoforms (α1 and α2), and the α1 subunit is ubiquitously expressed in all tissues, but the α2 subunit is predominantly expressed in skeletal and cardiac muscles [2, 3]. In response to energy limitation, increased cellular AMP or ADP directly interacts with the gamma subunit of AMPK and supports the kinase activity of the alpha subunit [4, 5]. Moreover, AMPK can also sense cellular glucose levels independently of cellular adenine nucleotide levels [6]. Recent studies demonstrated that aldolase, a key enzyme in the glycolysis pathway that catalyzes the conversion of fructose-1,6-bisphosphate (FBP), acts as a glucose metabolite-sensing protein that activates AMPK. Under glucose-limited conditions, FBP-unoccupied aldolase stimulates the recruitment of LKB1, a key upstream kinase of AMPK, to the lysosome, where the LKB1 directly phosphorylates and activates AMPK [7].

Given the critical role of AMPK in cellular catabolic processes, it has been postulated that reduced AMPK activity contributes to the development of metabolic disorders such as diabetes and obesity, as well as their complications [8]. Diabetic kidney disease (DKD) is the leading cause of end-stage renal disease, which affects almost one-third of diabetic patients worldwide [9–11]. Loss of glomerular function and glomerular fibrosis are the final consequences of DKD [9]. While a plethora of pathogenic factors have been proposed to lead to renal cell dysfunction in DKD [9, 12–22], dysfunctions of the podocyte, a highly differentiated glomerular epithelial cell, play a pivotal role in its development. Furthermore, recent studies have proposed that reduced AMPK activity in podocytes under diabetic conditions contributes to the development of DKD [23–26]. In support of the above, compounds (e.g., metformin, resveratrol, AICAR, and other compounds activating AMPK through the beta subunit) that indirectly or directly activate AMPK activity mitigate podocyte injuries and other renal cell dysfunctions under a diabetes-mimetic cell culture system or in diabetic model animals [27–31]. However, most observations of reduced podocyte damage or the renoprotective effect of AMPK come from cell culture experiments and from mice treated with AMPK activators, which may have off-target actions and glucose-lowering effects. Thus, whether a reduction in AMPK activity in podocytes has an autonomous impact on the development of podocyte and glomerular dysfunction remains largely elusive. Interestingly, it has been demonstrated that glomerular podocytes express both α1 and α2 AMPK catalytic subunits, which have redundant functions [32–35]. Therefore, we generated podocyte-specific AMPK1/2 double knockout (pmut) mice to determine the role of AMPK in podocytes in glomerular function in vivo. Surprisingly, we found that AMPK is dispensable for podocyte growth, survival, and glomerular function under ad libitum-fed conditions, as pmut mice live as long as wild-type mice without manifesting any pathological alterations in their kidneys. However, under diabetic conditions, pmut mice display elevated collagen synthesis in podocytes and endothelial-to-mesenchymal transition in glomerular endothelial cells, and the cumulative effects lead to greater glomerular fibrogenesis than in wild-type diabetic mice. These observations indicate that, under normal conditions, AMPK plays little role in maintaining podocyte function or that other kinases can adequately compensate for its key functions. However, under diabetic conditions, the remaining AMPK activity in podocytes of wild-type mice does play a renoprotective role by mitigating podocyte phenotypic changes and related glomerular endothelial activation.

## Results

### AMPK is dispensable for maintaining podocyte and glomerular function during normal development and aging

**Fig. 1:**
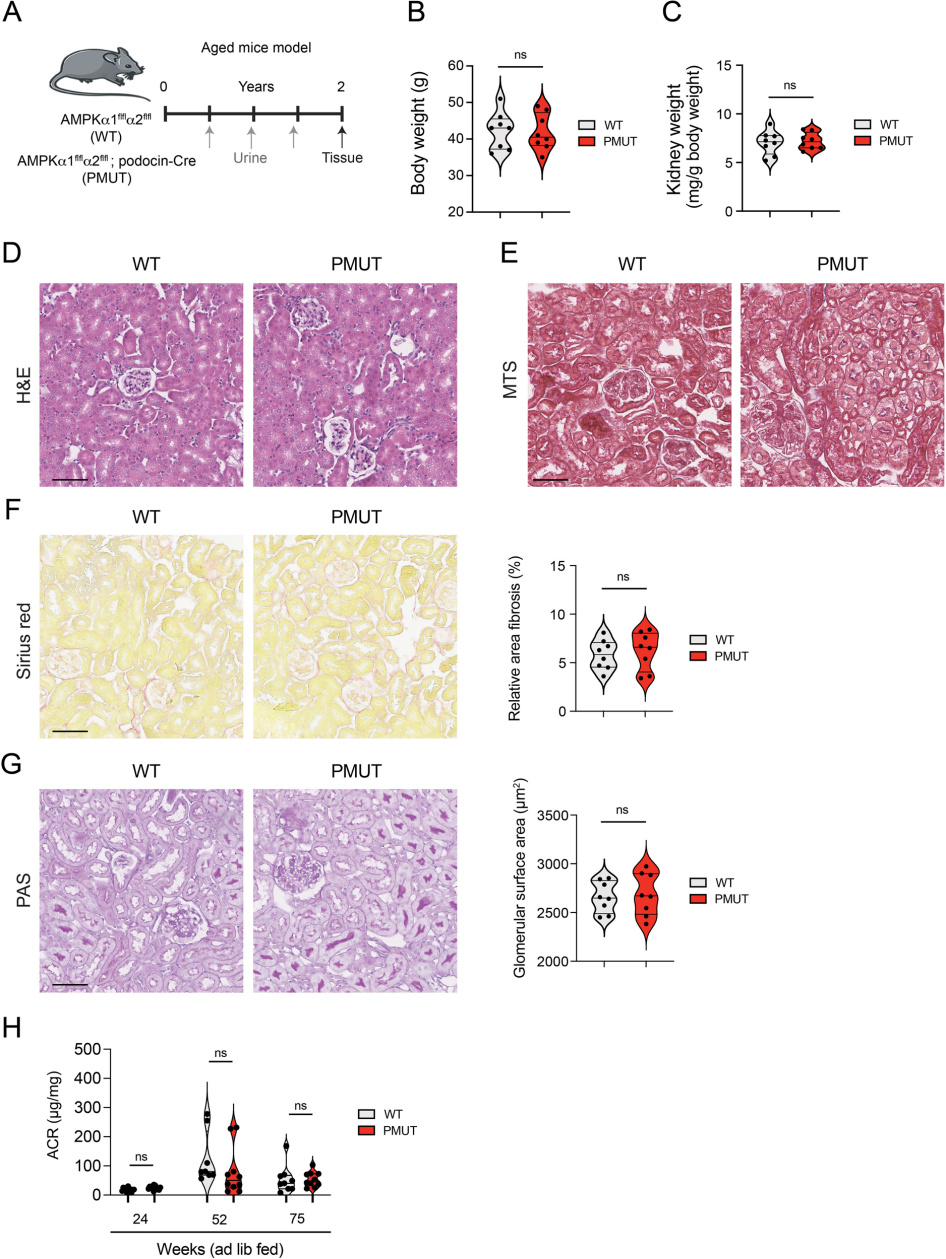
AMPK is dispensable for maintaining podocyte and glomerular function during normal development and aging. **A** A schematic diagram showing the experimental design analyzing the aged *Ampk α1* ^*fl/fl*^*α2* ^*fl/fl*^ mice (WT) and *Ampk α1* ^*fl/fl*^*α2* ^*fl/fl*^, *podocin-Cre* mice (pmut). WT and pmut mice were kept on a normal chow diet and had free access to water. At 2 years of age, mice were used for histopathological analyses. **B** Measurement of body weight. Data are shown as mean±SEM. ns denotes no significance in the statistical analysis. *N* = 8 was analyzed in/group. **C** Kidney weight/body weight was measured. Data are shown as mean±SEM. ns denotes no significance. *N* = 8 was analyzed in/group. **D**, **E** Hematoxylin-eosin and Masson-Trichrome staining were shown. **F** Sirius red staining was shown. The relative area of glomerular fibrosis was quantified. Data are shown as mean±SEM. ns denotes no significance. *N* = 8 was analyzed/group. **G**. Periodic Acid-Schiff (PAS) staining was shown. The glomerular surface area was calculated. The scale bar indicates 50 µm. Data are shown as mean ± SEM. ns denotes no significance. *N* = 8 was analyzed/group. **H**. Albumin-to-creatine ratio (ACR: µg/mg) was shown. 24-h urine samples were collected at the indicated time points, and the albumin and creatinine concentrations were determined. Data are shown as mean ± SEM. ns denotes no significance. *N* = 8 was analyzed/group.

To test the role of AMPK in podocytes, we generated podocyte-specific AMPK α1/α2 double knockout mutant mice (pmut) by crossing B6 background *Ampk α1* ^*fl/fl*^*α2* ^*fl/fl*^ mice with B6 background *podocin-Cre* mice (Fig. 1A). The isolated glomeruli of the pmut mice had significantly suppressed levels of both AMPKα1 and AMPKα2 mRNAs when compared to the *Ampk α1* ^*fl/fl*^*α2* ^*fl/fl*^ (WT) groups (Fig. S1A). The loss of the AMPK activity in podocytes was further analyzed by double staining of WT-1 (podocyte marker) and phosphorylated ULK1/ATG1 (S556), the site phosphorylated by AMPK, in the WT and pmut glomeruli [36]. Consistent with the reduction of AMPKα1 and AMPKα2 mRNAs in the glomeruli, levels of pATG1 were diminished in the podocytes of pmut mice (Fig. S1B). To determine the role of AMPK in regulating podocyte function, WT and the pmut mice were kept on free access to food and water for 2 years, and physiological parameters and kidney histology were monitored. The pmut mice (both male and female) remained healthy, similar to control WT mice, throughout the monitoring period. There was no significant difference in body weight, kidney weight, and blood glucose at 2 years old of pmut mice and littermate controls (Figs. 1B, C, and S1C). Histological analyses also demonstrated that no pathological alterations of glomerular and tubular structures (Hematoxylin-Eosin: HE), fibrosis (Masson Trichrome: MTS), or collagen deposition (Sirius red) were observed in the pmut mice cortex tissues compared to control mice (Fig. 1D, E, F). Periodic acid-Schiff (PAS) staining also showed no significant difference in carbohydrate accumulation or glomerular surface area between pmut and control mice (Fig. 1G). As expected, there were no apparent differences in urine albumin-to-creatinine ratios between the two groups (Fig. 1H).

### AMPK in podocytes is dispensable for reducing cellular mTORC1 activity and maintaining autophagy under normal development and aging conditions

**Fig. 2:**
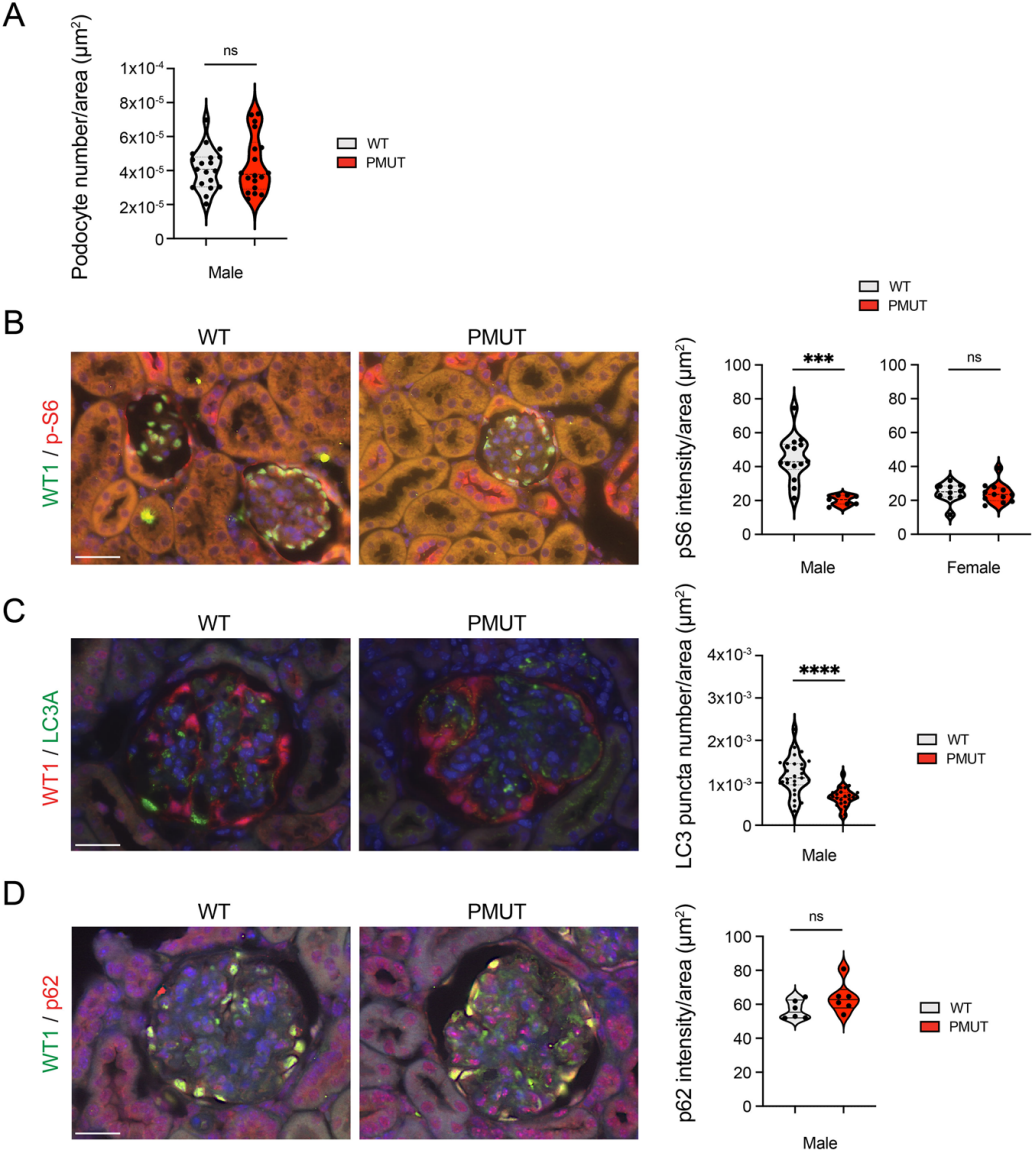
AMPK in podocytes is dispensable for reducing cellular mTORC1 activity and maintaining autophagy under normal development and aging conditions. **A**The number of WT1-positive podocytes per glomerulus (section) was determined in the indicated genotypes. Data are shown as mean±SEM. ns denotes no significance. *N* = 18–19 glomeruli from 5–7 mice per group were analyzed. **B** Levels of phosphorylated S6 were determined. Double staining for pS6 (S240/S244) and WT1 was shown in the indicated genotypes. The scale bar indicates 50 µm. The intensity of pS6 in the indicated glomeruli was quantified. Data are shown as mean±SEM, ***p < 0.001, *N* = 7–14. ns denotes no significance. **C** Double staining for WT1 and LC3 was shown. The number of LC3 puncta less than 8 µm^2^ in the indicated glomeruli was determined. Data are shown as mean±SEM. p****<0.0001. *N* = 6/group were analyzed. **D** Double staining for WT1 and P62 was shown. The intensity of p62 was normalized by glomerular area. The scale bar indicates 50 µm. Data are shown as mean±SEM. *N* = 6/group were analyzed. ns denotes no significance.

AMPK is known to act as a key kinase that protects cells against various metabolic stresses. However, the number of podocytes in the glomeruli of pmut mice was maintained and comparable to that in control mice at 2 years old (Fig. 2A). While AMPK has been proposed to act as a key suppressor for mTORC1 activity in various cells, including podocytes, levels of S6 phosphorylation, a downstream target of mTORC1-S6K1 activity, were not increased in the glomeruli of pmut mice compared to those in the glomeruli of wild-type mice (Fig. 2B). While levels of S6 phosphorylation in the glomeruli are similar between female WT and pmut mice, it was somewhat lower in the male pmut mice compared to that in the WT mice, suggesting that AMPK did not suppress mTORC1 activity under ad-lib fed conditions. Recent studies have proposed that, in contrast to the general paradigm, acute AMPK activation inhibits autophagy [37]. However, continuous AMPK activation still supports cellular autophagy by maintaining the expression of proteins involved in autophagy induction.

Furthermore, it has been reported that AMPK may play a key role in upregulating autophagy in podocytes [38]. While the number of LC3 puncta, which indicates an autophagosome, in the glomerulus of pmut mice was slightly decreased compared to that of WT mice, the podocytes of pmut mice still formed many autophagosomes (Fig. 2C). Consistently, there was no significant increase in the accumulation of p62 in pmut glomeruli compared to that in WT glomeruli (Fig. 2D), suggesting that basal autophagic activity was maintained in the podocyte lacking AMPK. This idea is also supported by the previous observations that loss of autophagy in podocytes resulted in age-dependent glomerulosclerosis and proteinuria, whereas the pmut mice did not display such pathological phenotypes [39]. These observations suggest that AMPK may not be a key regulator of mTORC1 and autophagy in podocytes under ad libitum feeding conditions, and that AMPK activity is dispensable for podocyte and glomerular function throughout the life of mice.

### AMPK loss in podocytes leads to enhanced glomerular fibrosis in a mouse model of type 1 diabetes

**Fig. 3:**
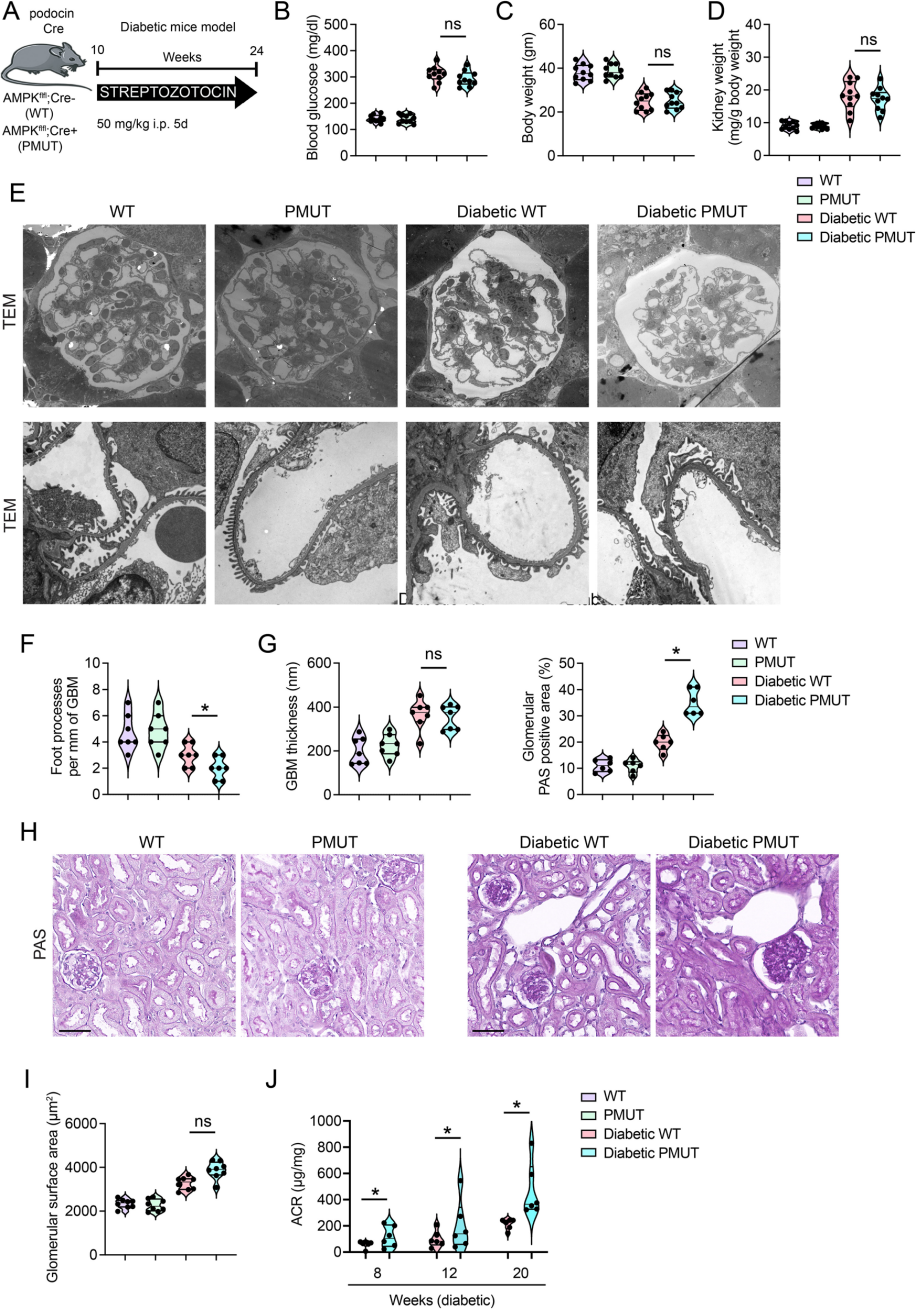
AMPK loss in podocytes leads to enhanced glomerular fibrosis in a mouse model of type 1 diabetes. **A** A schematic diagram showing the experimental design analyzing the diabetic *Ampk α1* ^*fl/fl*^*α2* ^*fl/fl*^ mice (WT) and *Ampk α1* ^*fl/fl*^*α2* ^*fl/fl*^, *podocin-Cre* mice (pmut). The indicated 10-week-old mice were treated with STZ at five low doses (50 mg/kg body weight). Diabetic mice were selected based on their blood glucose levels above 200 mg/dl, fed chow, and given free access to water for 24 weeks. 24-h urine collections were performed at the indicated time points, and tissues were analyzed 24 weeks after STZ treatment. At the end of the experiment, mice in each group were sacrificed, and their kidneys were examined histologically. **B**, **C**, **D** Blood glucose, body weight, and kidney weight/body weight were shown (24 weeks post-STZ treatment). Data are shown as mean±SEM. *N* = 10 was analyzed/group. ns denotes no significance. **E** Representative transmission electron microscopy images were shown. *N* = 3/group. **F**, **G** Foot process effacement and glomerular basement membrane thickness were analyzed using the TEM images, and quantification was calculated using ImageJ software. Seven independent images were analyzed. Data are shown as mean±SEM, *p < 0.01, *N* = 7(images from 3 mice). **H** Periodic Acid-Schiff (PAS) staining was shown in the indicated group. **I** Glomerular surface area was measured using the PAS images. The scale bar indicates 50 µm. Data are shown as mean±SEM. *N* = 6 was analyzed/group. **J** Albumin-to-creatine ratio (ACR) was determined by performing ELISA in diabetic WT and pmut mice. Data are shown as mean ± SEM, *p < 0.05, *N* = 6.

To investigate the role of AMPK in podocytes in regulating glomerular function under diabetic conditions, we induced diabetes in WT and pmut mice with Streptozotocin (STZ). Five low doses of STZ were injected intraperitoneally, and their glomerular function and histological alterations were monitored (Fig. 3A). Both WT and pmut diabetic animals showed similar levels of hyperglycemia, body weight loss, and higher kidney weights compared to non-diabetic animals (Fig. 3B, C, D). As evidenced by transmission electron microscopy, glomerular ultrastructure showed no significant differences between non-diabetic WT and pmut mice (Fig. 3E). Both diabetic control and pmut mice displayed foot process effacement and GBM thickening (Fig. 3E, F, G). While levels of GBM thickening in diabetic WT and pmut mice were similar, the foot process effacement of pmut podocytes was slightly more severe than that of control podocytes under diabetic conditions (Fig. 3F, G). Furthermore, histological analyses showed slightly increased glomerular size in pmut diabetic mice compared to WT diabetic mice (Fig. 3H, I). Consistent with these observations, pmut diabetic mice displayed higher urine albumin-to-creatinine ratios (ACR) than diabetic WT mice (Fig. 3J). These observations suggest that AMPK plays an important role in preventing podocyte dysfunction in these diabetic model animals.

**Fig. 4:**
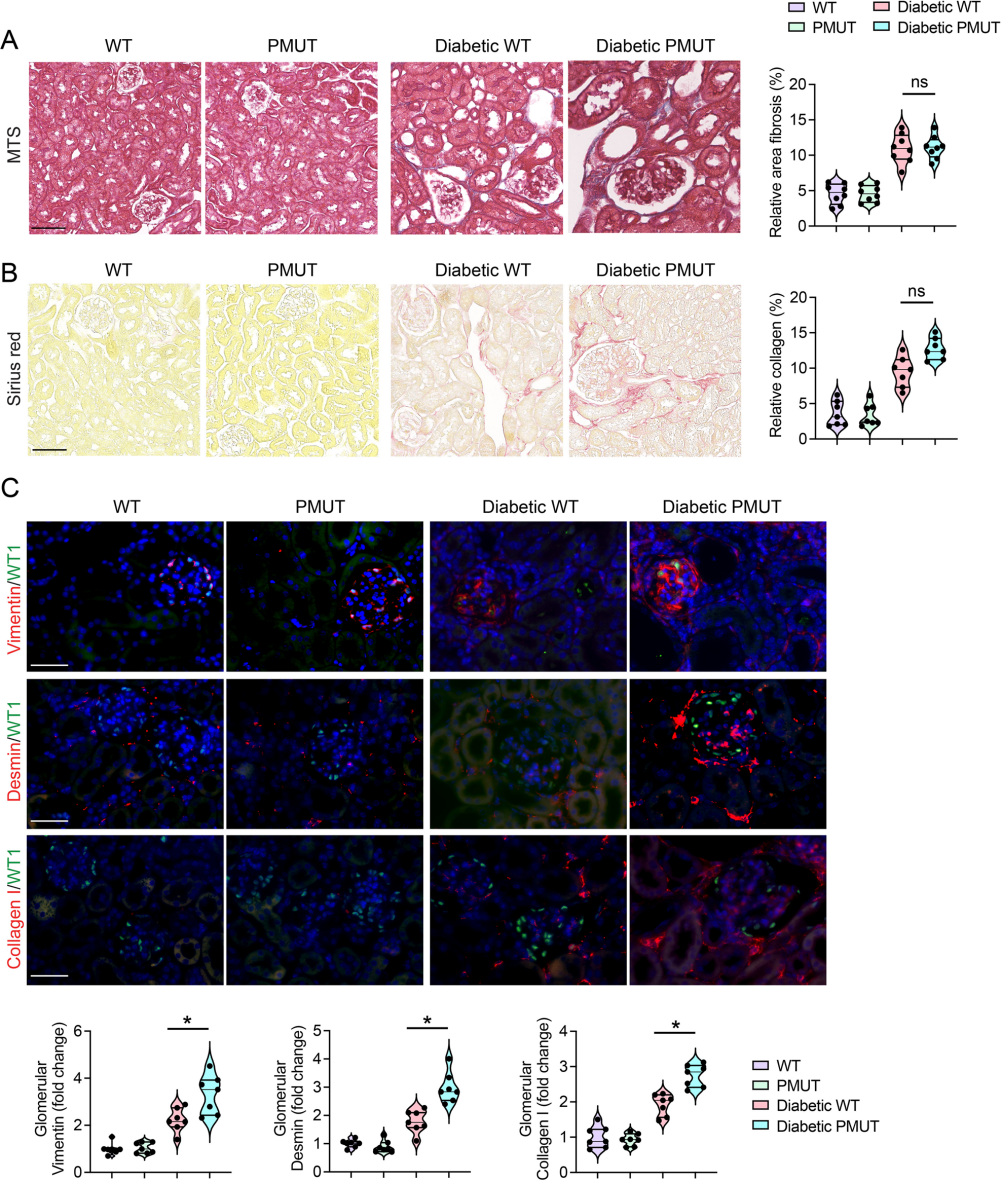
AMPK loss in podocytes leads to enhanced glomerular fibrosis in a mouse model of type 1 diabetes (continued). **A** Masson Trichrome staining (MTS) was performed, and levels of fibrosis in the interstitial region were quantified. The scale bar indicates 50 µm. Data are shown as mean ± SEM. *N* = 10 was analyzed/group. **B** Sirius red staining was performed, and levels of collagen deposition in the interstitial region were quantified. The scale bar indicates 50 µm. Data are shown as mean±SEM. *N* = 10 was analyzed/group. **C** Double immunofluorescence staining of vimentin, desmin, or collagen I with WT1 was shown. The scale bar indicates 50 μm. Representative images are shown. Data are shown as mean±SEM, *p < 0.05, *N* = 7.

To investigate further pathological alterations in pmut diabetic mice, we performed Masson’s trichrome and Sirius red staining to evaluate tissue fibrosis. These assays demonstrated an increased collagen deposition in mesangial and tubulointerstitial areas in both diabetic groups compared to non-diabetic groups. There was no difference in levels of interstitial fibrosis between the diabetic groups (Figs. 4A and 4B). However, consistent with the observations in PAS staining (Fig. 3H, J), immunofluorescence studies revealed a higher expression of mesenchymal proteins, such as vimentin and desmin, and a higher level of collagen I deposition in the glomeruli of diabetic pmut mice compared to those of WT diabetic mice, indicative of higher glomerular fibrosis in diabetes pmut mice (Fig. 4C). We also monitored the macrophage polarization in WT and pmut non-diabetic and diabetic kidneys. While levels of CD206, a marker of M2 macrophage, were lower, levels of iNOS, a marker of M1 macrophage, were higher in the diabetic kidney compared to the non-diabetic kidney. In addition, iNOS expression was significantly higher in the kidneys of pmut diabetic mice than in diabetic WT mice, suggesting that loss of AMPK in podocytes is associated with pro-inflammatory macrophage accumulation in the diabetic kidney (Fig. S2).

**Fig. 5:**
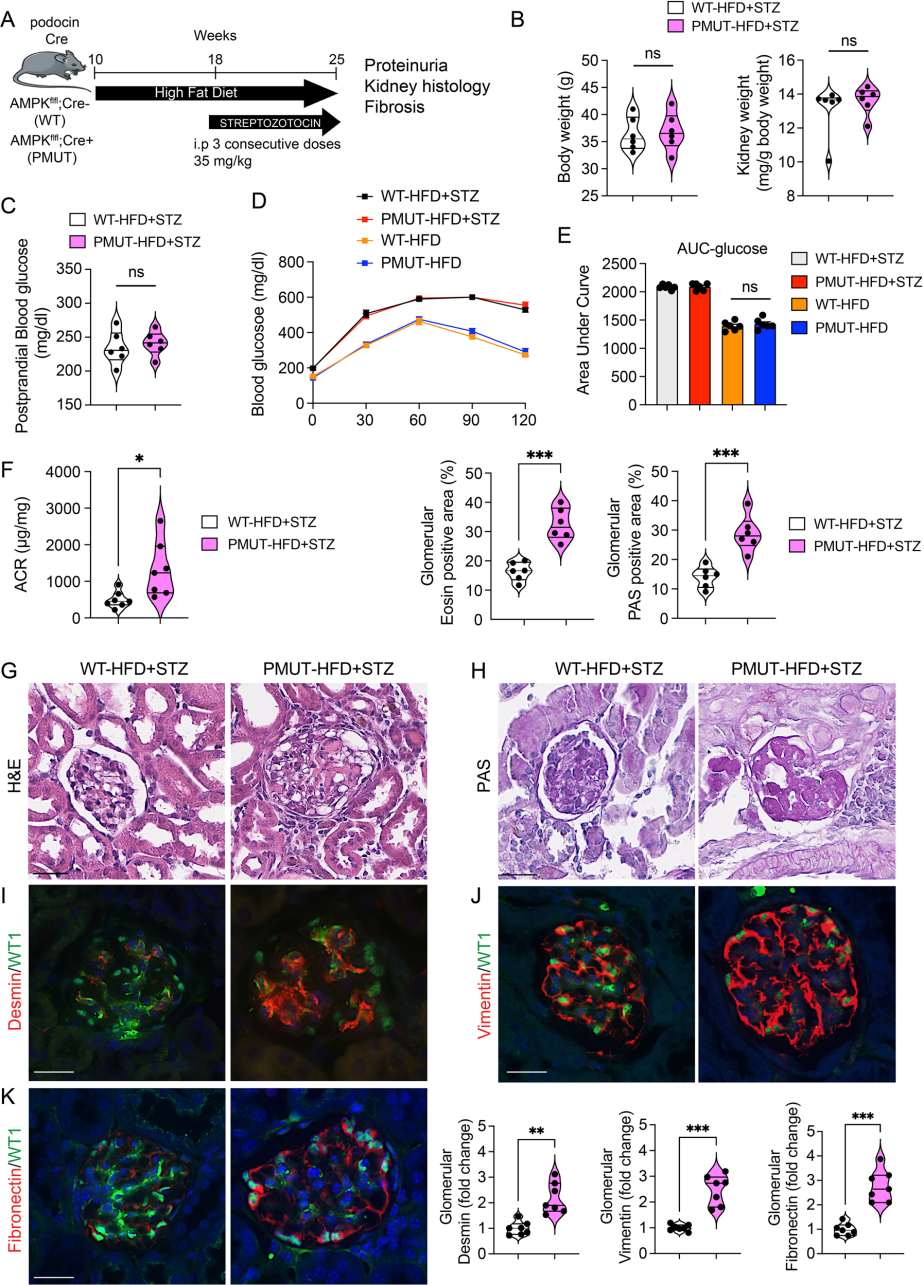
AMPK loss in podocytes leads to a fibrotic phenotype in a mouse model of type 2 diabetes (HFD + STZ). **A** Schematic chart showing the development of HFD-fed STZ-injected WT and pmut mice. HFD was fed for 8 weeks, and then three consecutive STZ injections (35 mg/kg) were given to these mice. After 7 weeks of STZ, mice were analyzed for IPGTT, proteinuria, and fibrotic complications in the kidneys of WT and pmut mice. **B** Body weight and kidney weight. Data are shown as mean±SEM, *N* = 6. n.s. indicates no significance. **C** Postprandial blood glucose, *N* = 6/group, was analyzed. **D** IPGTT was analyzed. A 1 g glucose load was given to these mice. Blood glucose was analyzed at 0, 30, 60, 90, and 120 min post glucose injection. *N* = 6/group was analyzed. **E** AUC-Glucose was analyzed from IPGTT glucose values. *N* = 6/group was analyzed. **F** Albumin-to-creatinine ratio was analyzed. Data are shown as mean ± SEM, *p < 0.05, *N* = 7. **G** Representative images, hematoxylin-eosin (H&E), are shown. The scale bar is 50 µm. Glomerular Eosin-positive areas were quantified. Data are shown as mean ± SEM, ***p < 0.001, *N* = 6. **H** PAS staining was analyzed in the kidneys of both genotypes. Representative images are shown. The scale bar is 50 µm. Glomerular PAS-positive areas were determined. Data are shown as mean±SEM, ***p < 0.001, *N* = 6. **I**–**K** Double immunofluorescence staining with WT1 and desmin, vimentin, or fibronectin, in the indicated glomeruli is shown. Representative images are shown. The scale bar is 50 µm. The signal intensity of desmin, vimentin, and fibronectin in the indicated glomerulus was quantified. Data are shown as mean±SEM. **p < 0.01, ***p < 0.001, *N* = 6.

We further analyzed the role of AMPK in podocytes under type 2 diabetic conditions using the STZ/high-fat diet mouse model (Fig. 5A). Consistent with observations in the type 1 diabetic mouse model, there were no remarkable alterations in body weight, kidney weight, postprandial blood glucose, or glucose tolerance; however, albuminuria levels were significantly higher in the diabetic pmut mice compared to WT diabetic mice (Fig. 5B–F). H&E staining revealed more serious glomerular damage, and PAS displayed higher sclerotic and fibrotic glomeruli in diabetic pmut mice compared to the WT diabetic mice (Fig. 5G, H). Higher desmin, vimentin, and fibronectin accumulations in the glomeruli of pmut diabetic mice compared to WT diabetic mice were observed, supporting the idea that AMPK protects diabetic glomeruli from pathological fibrotic changes (Fig. 5I–K).

To test whether AMPK exerts an antifibrotic effect under diabetic conditions, we examined the impact of the AMPK activator on the expression of collagen I and fibronectin in cultured podocytes under high glucose and normoglycemic conditions (Fig. S3A). As expected, high glucose reduced AMPK phosphorylation in cultured podocytes, whereas it increased collagen 1 and fibronectin expression (Fig. S3B). The enhanced expression of collagen 1 and fibronectin was attenuated by the treatment with the AMPK activator (Fig. S3B). Under high-glucose culture conditions, levels of collagen I, TGFβR1, IL-1β, IL-6, and NF-κB1 mRNA were also increased, which were effectively suppressed by AMPK activator treatment in cultured podocytes (Fig. S3C and S3D).

We also examined whether metformin, an anti-diabetic drug that stimulates AMPK, protects against glomerular fibrosis in STZ-induced diabetic mice. Consistent with previous observations, metformin treatment enhanced AMPK phosphorylation and reduced fibrotic complications in diabetic glomeruli [40–43] (Fig. S4).

-

### AMPK loss in podocytes alters lipid and glucose metabolism in a mouse model of type 1 diabetes

**Fig. 6:**
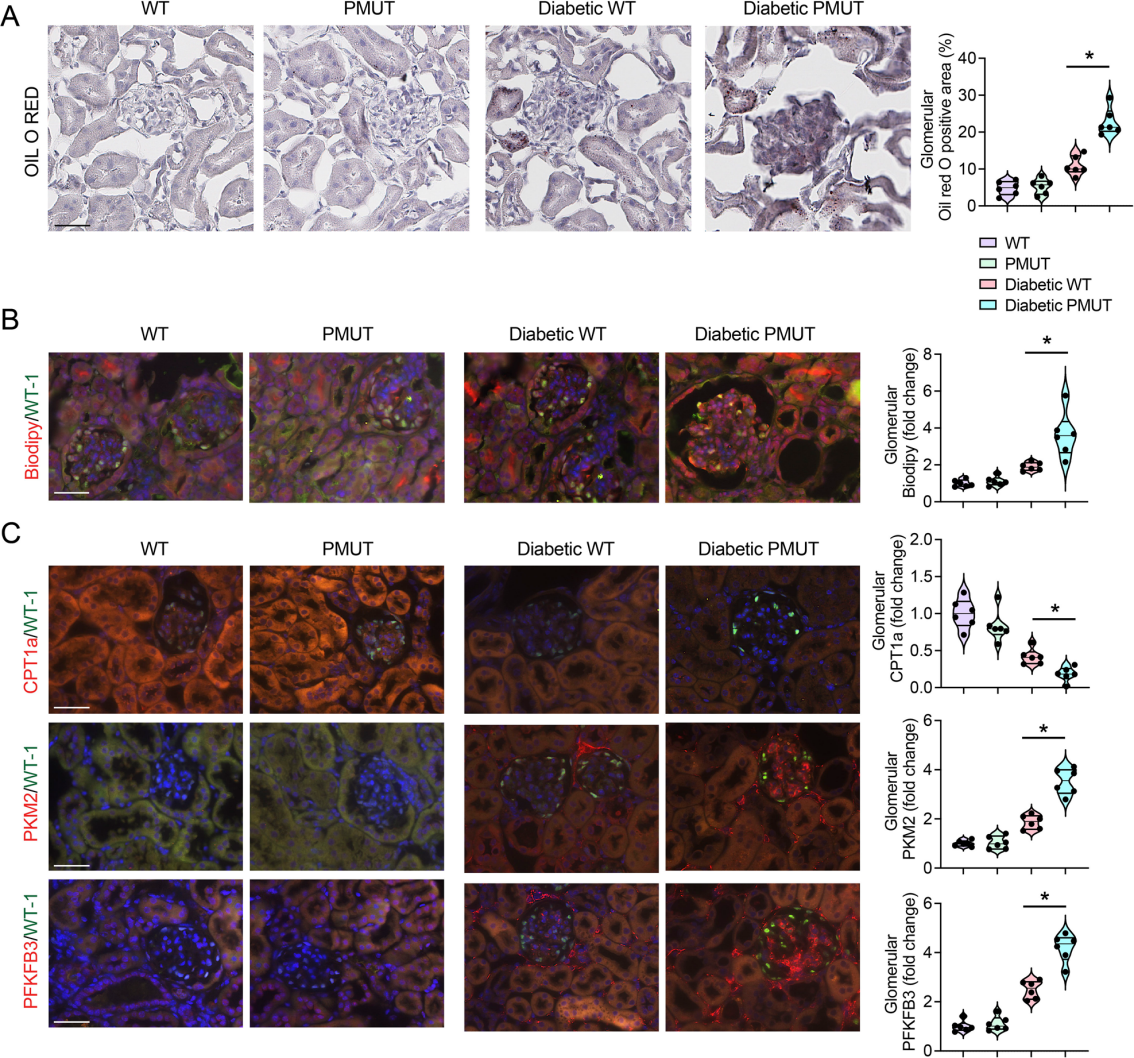
AMPK loss in podocytes alters lipid and glucose metabolism in a mouse model of type 1 diabetes. **A** Oil O Red staining was performed in non-diabetic and diabetic WT and pmut mice. Representative microphotographs are shown. The scale bar indicates 50 µm. *N* = 6 was analyzed per group. Data are shown as mean ± SEM, *p < 0.05. **B** Double immunofluorescence staining of Biodipy and WT1 was performed in the indicated genotypes. The scale bar indicates 50 µm. Representative images are shown. Data are shown as mean±SEM, *p < 0.05, *N* = 6. **C** Double immunofluorescence staining of CPT1a, PFKFB3, or PKM2 with WT-1 was performed. The scale bar indicates 50 µm. Representative images are shown. Data are shown as mean ± SEM, *p < 0.05, *N* = 6.

Metabolic shifts play critical roles in kidney cell health and disease processes, and recent studies suggest that alterations in fuel preference are associated with fibrogenesis in kidney cells [44–53]. AMPK is a well-known kinase that breaks down lipids to generate energy for cell survival. We examined lipid accumulation in kidney cells using Oil Red O and Bodipy staining. While there was no remarkable difference in lipid deposition in the kidney of non-diabetic WT and pmut mice (Figs. 6A and S1D), overall, Oil-red O-positive tissue levels were increased under diabetic conditions. Notably, the glomeruli of diabetic pmut mice displayed a higher level of lipid deposition compared to the glomeruli of diabetic WT mice (Fig. 6A). Immunofluorescent staining of Biodipy, which stains most of the neutral lipids, also revealed no remarkable difference in lipid deposition in the kidneys of non-diabetic WT and pmut mice. However, consistent with the results of Oil-red O staining, the diabetic pmut mice displayed higher levels of Biodipy-positive podocytes in the diabetic pmut mice compared to diabetic WT mice (Fig. 6B). Moreover, levels of carnitine palmitoyltransferase 1a (CPT1a), a key enzyme in fatty acid oxidation (FAO), were largely reduced in diabetic kidney tissues in both genotypes (Fig. 6C). Furthermore, levels of CPT1a expression in the podocytes of pmut diabetic mice were lower than those in WT diabetic mice (Fig. 6C), suggesting that the podocytes in diabetic pmut mice may have a more significant limitation in utilizing lipids and generating cellular energy from FAO. We also monitored levels of key enzymes in the glycolysis pathway. Under diabetic conditions, levels of 6-phosphofructo-2-kinase/fructose-2,6-biphosphatase 3 (PFKFB3) and pyruvate kinase muscle type 2 (PKM2) expression in podocytes were increased in both genotypes compared to those in the podocytes of non-diabetic mice. Interestingly, enhanced expression of these glycolytic enzymes was more pronounced in glomerular cells, including podocytes, of diabetic pmut mice (Fig. 6C). While AMPK has been recognized as a key stimulator of glycolysis in response to metabolic stress, these observations suggest that loss of AMPK in podocytes may further enhance glycolysis to generate cellular fuel in podocytes and other glomerular cells under type 1 diabetic conditions.

### AMPK loss in podocytes facilitates endothelial-to-mesenchymal transition in the mouse model of type 1 diabetes

**Fig. 7:**
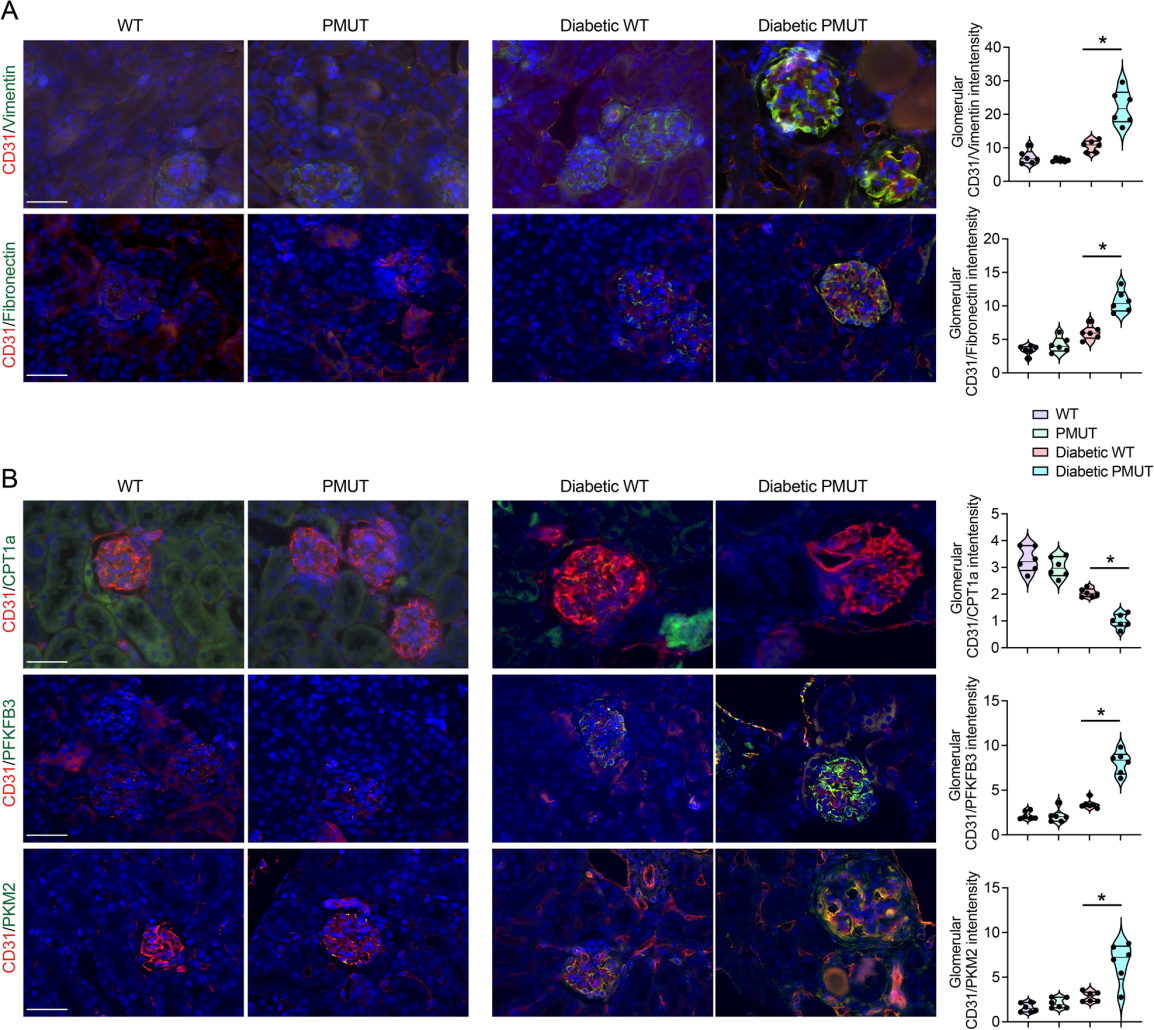
AMPK loss in podocytes facilitates endothelial-to-mesenchymal transition in the mouse model of type 1 diabetes. **A** Endothelial-to-mesenchymal transition was determined by double immunofluorescence staining of vimentin or fibronectin with CD31. Representative immunofluorescence images are shown here. The scale bar indicates 50 µm. *N* = 6 was analyzed/group. Data are shown as mean ± SEM, *p < 0.05. **B** Double immunofluorescence staining of CPT1a, PFKFB3, or PKM2 with CD31 was performed. The scale bar indicates 50 µm. Representative images are shown. Data are shown as mean±SEM, *p < 0.05, *N* = 6.

Endothelial-to-mesenchymal transition (EndMT) plays a key role in the development of glomerular fibrosis [54–61]. Since we observed that diabetic pmut mice displayed more glomerular fibrosis than diabetic control mice, we investigated whether loss of AMPK in podocytes facilitates EndMT in the glomerular endothelial cells under diabetic conditions. Double immunofluorescence staining with CD31, an endothelial marker, and vimentin or fibronectin revealed that higher levels of vimentin or fibronectin expression in the CD31-positive endothelial cells in the glomeruli of diabetic mice compared to those of non-diabetic mice. Importantly, this effect was more pronounced in the glomeruli of diabetic pmut mice than in those of diabetic control mice (Fig. 7A), indicating that loss of AMPK in podocytes facilitates EndMT of glomerular endothelial cells under diabetic conditions. Impairment of normal lipid or glucose metabolism has been linked to the EndMT phenotype in endothelial cells [44, 45, 62]. Therefore, we also analyzed levels of CPT1a and glycolytic enzymes in the endothelial cells. Consistently, overall levels of CPTa expression were reduced in the kidney tissues of diabetic groups compared to non-diabetic counterparts (Fig. 7B). There was a consistent trend toward greater reduction in CPT1a levels in the glomeruli and CD31-positive endothelial cells of diabetic pmut mice compared with diabetic control mice (Fig. 7B). In contrast, levels of PFKFB3 and PKM2 expression were again enhanced in the glomeruli of diabetic mice compared to those of non-diabetic mice, and their expression in CD31-positive endothelial cells was much higher in the diabetic pmut mice compared to those in diabetic control mice (Fig. 7B). These observations indicate that loss of AMPK in the podocytes also leads to similar metabolic derangements in the glomerular endothelial cells and facilitates EndMT under diabetic conditions.

### Loss of AMPK in podocytes causes c-GAS-STING-mediated inflammation

**Fig. 8:**
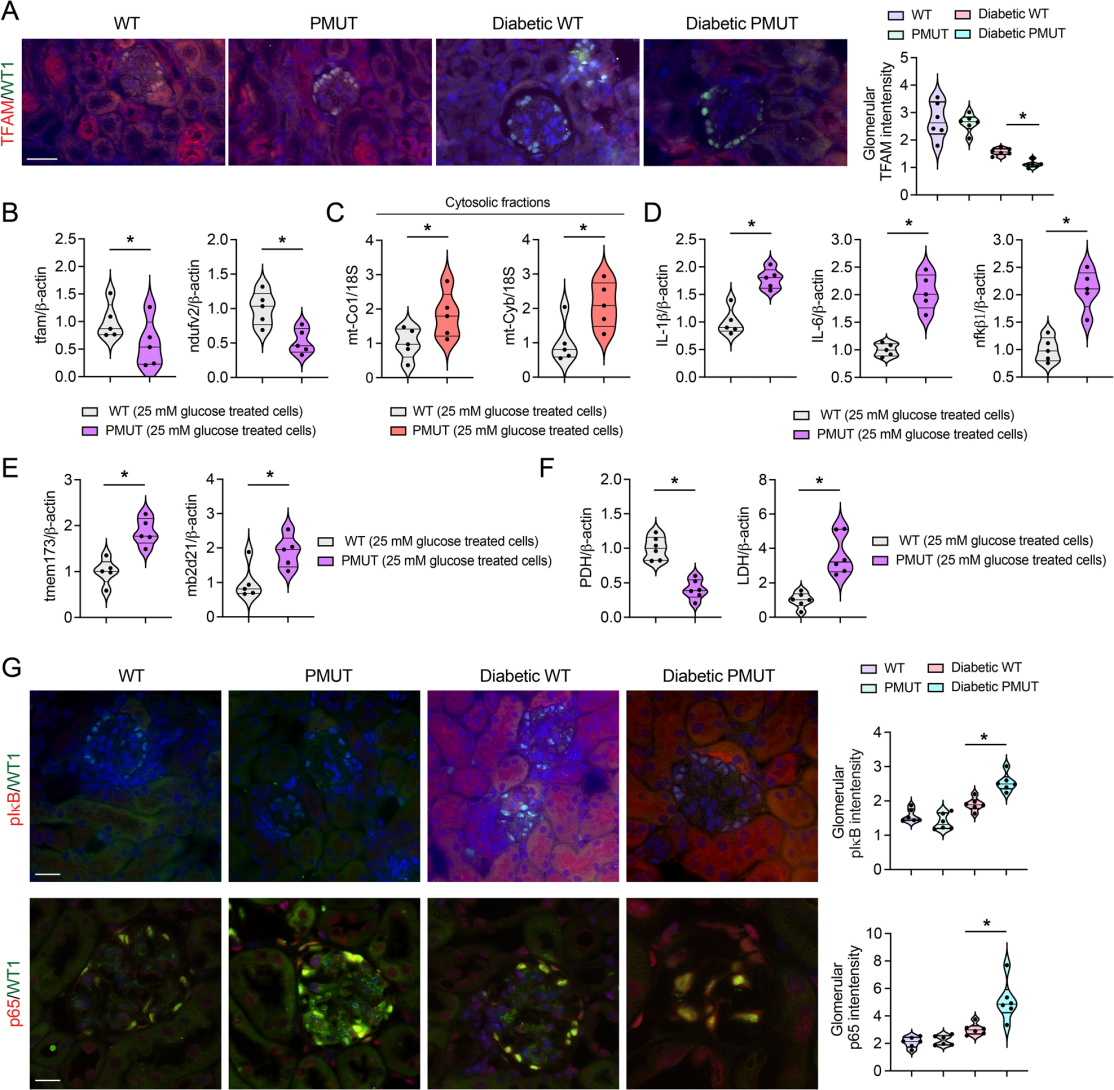
Loss of AMPK in podocytes causes c-GAS-STING-mediated inflammation. **A** Double immunofluorescence staining of TFAM and WT-1 was shown in the non-diabetic and diabetic WT and pmut mice. The scale bar indicates 50 µm. Representative images are shown. *N* = 6 was analyzed/group. The intensity of TFAM in the indicated glomeruli was quantified. Data are shown as mean ± SEM, *p < 0.05. **B** Quantitative qPCR analyses of tfam and ndufv2 transcripts in the indicated glomeruli were shown. mRNAs were extracted from the purified glomeruli from WT or pmut mice under high-glucose (25 mM) culture conditions (for 96 hours). Data are shown as mean ± SEM. *N* = 6, *p < 0.05. **C** Quantitative qPCR analyses of mt-Co1 and mt-Cyb transcripts in the cytosolic fractions of high-glucose (25 mM) treated cultured glomeruli from WT and Pmut mice. 18S was used as an internal control. Data are shown as mean ± SEM. *N* = 6, *p < 0.05. **D**, **E** Quantitative qPCR analyses of *Il-1β, Il-6, Nfkb1, Tmem173*, and *Mb2d21* transcripts in the indicated glomeruli cultured in high-glucose (25 mM) media. Data are shown as mean ± SEM., *N* = 6, *p < 0.05. **F** Quantitative qPCR analyses of *Phd* and *ldh* transcripts in the indicated glomeruli cultured in high-glucose (25 mM) media. Data are shown as mean ± SEM. *N* = 6, *p < 0.05. **G** Double immunofluorescence staining of p-Iκβ or p65 with WT1 was shown in non-diabetic and diabetic WT and pmut mice. The scale bar indicates 50 µm. Representative images are shown. *N* = 6 was analyzed/group. Data are shown as mean ± SEM, *p < 0.05.

Metabolic reprogramming, such as defective normal lipid metabolism and associated lipotoxicity, is detrimental to cells and their organelles, including mitochondria [63, 64]. Damaged mitochondria often release mitochondrial DNA into the cytoplasm, triggering the activation of the cytosolic GMP-AMP synthase (cGAS) and the stimulator of interferon genes (STING) pathway, a critical innate immune response [65, 66]. The activation of this pathway enhances the transcription of pro-inflammatory genes [63, 65]. To investigate mitochondrial integrity and expression in diabetic pmut mice, we monitored levels of mitochondrial transcription factor A (TFAM). Overall, TFAM expression levels were decreased in the kidney tissues of both genotypes under diabetic conditions. Importantly, the glomeruli and podocytes of diabetic pmut mice displayed reduced levels of TFAM compared to those of control diabetic mice (Fig. 8A). To test whether these observations can be recapitulated in the in vitro system, glomeruli were isolated from the kidney cortex tissue of pmut and control mice (10-week-olds) and cultured in high-glucose media. Consistently, levels of *Tfam* and *Ndufv2* mRNAs were lower in the glomeruli of pmut mice compared to those of control mice (Fig. 8B). To test if loss of AMPK leads to reduced mitochondrial integrity, we monitored levels of *mt-Co1* and *mt-Cyb* transcripts in the cytosolic fractions of cultured glomeruli. Levels of cytosolic *mt-Co1* and *mt-Cyb* transcripts were increased in the glomeruli of pmut mice compared with those in control mice, suggesting greater leakage of mitochondrial components from pmut glomerular cells under high-glucose culture conditions (Fig. 8C). Furthermore, levels of pro-inflammatory gene expression, such as *IL-1β*, *IL-6*, and *NF-*κ*B1*, were significantly higher in glomeruli from pmut mice than those from control mice under high-glucose culture conditions (Fig. 8D). The gene expression of *Mb21d2* (cGAS) and *Tmem173* was upregulated by various transcriptional factors, including NF-κB and STAT1 [63, 65, 67]. Consistently, levels of *Mb21d2 and Tmem173* were higher in the glomeruli of pmut mice than those in control mice (Fig. 8E), suggesting that loss of AMPK predisposes to augment mitochondrial-derived inflammation through the cGAS-STING pathway under high glucose conditions. We also observed that the isolated glomeruli from the pmut mice expressed a higher level of lactate dehydrogenase (LDH) mRNA expression, whereas lower levels of pyruvate dehydrogenase (PDH) mRNA expression compared to control glomeruli under high glucose culture conditions (Fig. 8F), suggesting that loss of AMPK in podocytes may also lead to a metabolic shift in the glycolytic pathway to produce lactate, which has been found to accelerate the fibrotic process in many cell types [27, 68–72].

Finally, immunofluorescent staining demonstrated that levels of phospho-IκB were generally higher in diabetic kidney tissues, including glomeruli and tubular cells in both groups, and the intensity was higher in the glomeruli of diabetic pmut mice than in those of control diabetic mice (Fig. 8G). Accordingly, nuclear p-65 expression accumulated more in glomerular cells, especially podocytes, of diabetic pmut mice compared to those of control diabetic mice (Fig. 8G). These observations suggest that loss of AMPK leads to lipid accumulation, mitochondrial damage, and activation of the cGAS-STING pathway, resulting in inflammation in type 1 diabetic mice.

## Discussion

Podocytes are highly differentiated epithelial cells. While they lack the replication capacity, they remain metabolically active to maintain the functions of the filtration barrier and the glomerular basement membrane by operating high levels of both anabolic (e.g., protein synthesis) and catabolic (e.g., autophagy) processes. AMPK is known to function as a central regulator that stimulates catabolic processes in various cells, including podocytes. Many previous reports have indicated that reduced AMPK activity plays a key role in the development of DKD, based on two categories of biological observations. Firstly, AMPK activity, which is inhibited by both cellular energy (e.g., ATP) and glucose metabolites (e.g., FBP), is generally reduced in the diabetic kidney, where glomerular cells are exposed to high levels of glucose and nutrients [6, 73]. Second, diabetic mice treated with compounds that directly or indirectly enhance AMPK activity display some renoprotective effects against DKD development in several animal models [27, 28, 72, 74–80]. Furthermore, these AMPK activators reduce podocyte vulnerability to metabolic stressors associated with diabetes (e.g., high glucose) in a cell culture system. In addition to these observations, aberrant mTORC1 activation in podocytes has been observed in diabetic animals and patients, leading to pathological podocyte hypertrophy and detachment from the GBM, and is thought to contribute to the development of DKD [81–83]. Importantly, AMPK acts as a key suppressor of mTORC1 and may enhance autophagy (at least in long-term AMPK activation) in various types of cells [37, 84–86], including cultured podocytes, supporting the idea that reduced AMPK activity in podocytes under diabetic conditions may underlie the pathogenesis of podocyte dysfunction and DKD. If this idea is correct, ablation of AMPK in podocytes likely leads to podocyte vulnerability and injury in an autonomous manner and may manifest DKD-like phenotypes, as seen in the model in which mTORC1 activity is genetically activated specifically in podocytes [82, 87]. However, no report has been made on the glomerular phenotypes of podocyte-specific AMPK knockout mice, in which both α1 and α2 catalytic subunits are ablated. Hence, we report here that, contrary to our prediction, AMPK is dispensable for podocyte development and its function during normal development and aging, as pmut KO mice do not exhibit any pathological glomerular alterations at 2 years old compared to their control littermates. These observations were somewhat unexpected, given AMPK’s reported key roles in maintaining energy homeostasis, metabolic processes, and cytoskeletal organization. Intriguingly, levels of mTORC1 activity were not significantly increased in the podocytes of pmut mice compared with those in control mice under normal ad libitum feeding conditions. Furthermore, there is no obvious impairment of autophagy in the podocytes of pmut mice. These observations suggest that AMPK is likely inactive in podocytes under normal developmental conditions, or that other pathways can fully compensate for its functions in maintaining podocytes. It has been reported that the activity of autophagy in podocytes was not inhibited by canonical mTORC1 activity, as autophagy is still active in the podocytes lacking the functional TSC complex, in which canonical mTORC1 is highly active [88]. The study proposed that AMPK plays a key role in maintaining autophagy in podocytes. However, our study suggests that under normal physiological conditions, AMPK is also dispensable for maintaining autophagy activity in podocytes. While AMPK activation provides beneficial effects on stressed podocytes, the podocytes in mice can maintain their healthy growth and essential filtration and other glomerular functions without AMPK.

It has been postulated that AMPK activity is reduced in many tissues, including kidney cells (e.g., tubular cells and podocytes), in both type 1 and type 2 diabetic animals [27, 72, 89–97]. Our genetic studies suggested that the remaining reduced AMPK activity plays an important role in mitigating the progression of podocyte and glomerular dysfunction, especially under diabetic conditions. Mechanistically, we demonstrated that the remaining AMPK activity in podocytes is critical for mitigating lipid accumulation, mitochondrial damage, and inflammation, as well as the phenotypic changes in glomerular endothelial cells. Consistent with the well-known role of AMPK in stimulating lipid catabolism, loss of AMPK led to lipid accumulation in podocytes in type 1 diabetes, under which podocytes may take up more FFAs from the filtration area. The reduction in CPT1a expression further exaggerates cytosolic lipid retention and likely causes lipotoxicity in podocytes. Interestingly, in contrast to the paradigm that AMPK stimulates glycolysis under metabolically stressed conditions, we observed elevated expression of essential glycolytic enzymes, such as PFKFB3 and PKM2, which promote glycolysis to generate pyruvate in podocytes and other glomerular cells, including endothelial cells, in diabetic pmut mice. These observations suggest that loss of AMPK in podocytes leads to a metabolic shift that favors glycolysis for fuel generation in glomerular cells under type 1 diabetic conditions. Furthermore, the isolated glomeruli from the pmut mice showed higher levels of LDH transcript and lower levels of PDH expression compared to control glomeruli under high-glucose culture conditions, suggesting a tendency toward lactate production, which has been found to accelerate the fibrotic process in many cell types [98–103]. Interestingly, our observations indicate that loss of AMPK in podocytes accelerated mesenchymal activation (endothelial-to-mesenchymal transition, EndMT) in glomerular endothelial cells under diabetic conditions. The glomerular endothelial cells in the diabetic pmut mice also displayed greater glycolytic enzyme and lower CPT1a expression than those in control diabetic mice, suggesting a metabolic shift in the endothelial cells. Under normal filtration conditions, glomerular endothelial cells provide the essential signals that maintain podocyte health and physiological functions. In contrast, hyperfiltration that occurs under diabetic conditions is thought to disrupt critical endothelial-podocyte crosstalk, resulting in the failure of glomerular functions [104–106]. It is postulated that podocytes also support endothelial cell health. Previous studies suggested that podocyte dysfunction increases basement membrane permeability, which may underlie mechanisms by which podocyte dysfunction can alter endothelial cell metabolism under diabetic conditions [107]. Further studies will be required to investigate the underlying mechanisms and factors that facilitate EndMT upon AMPK inhibition in the podocytes.

**Fig. 9:**
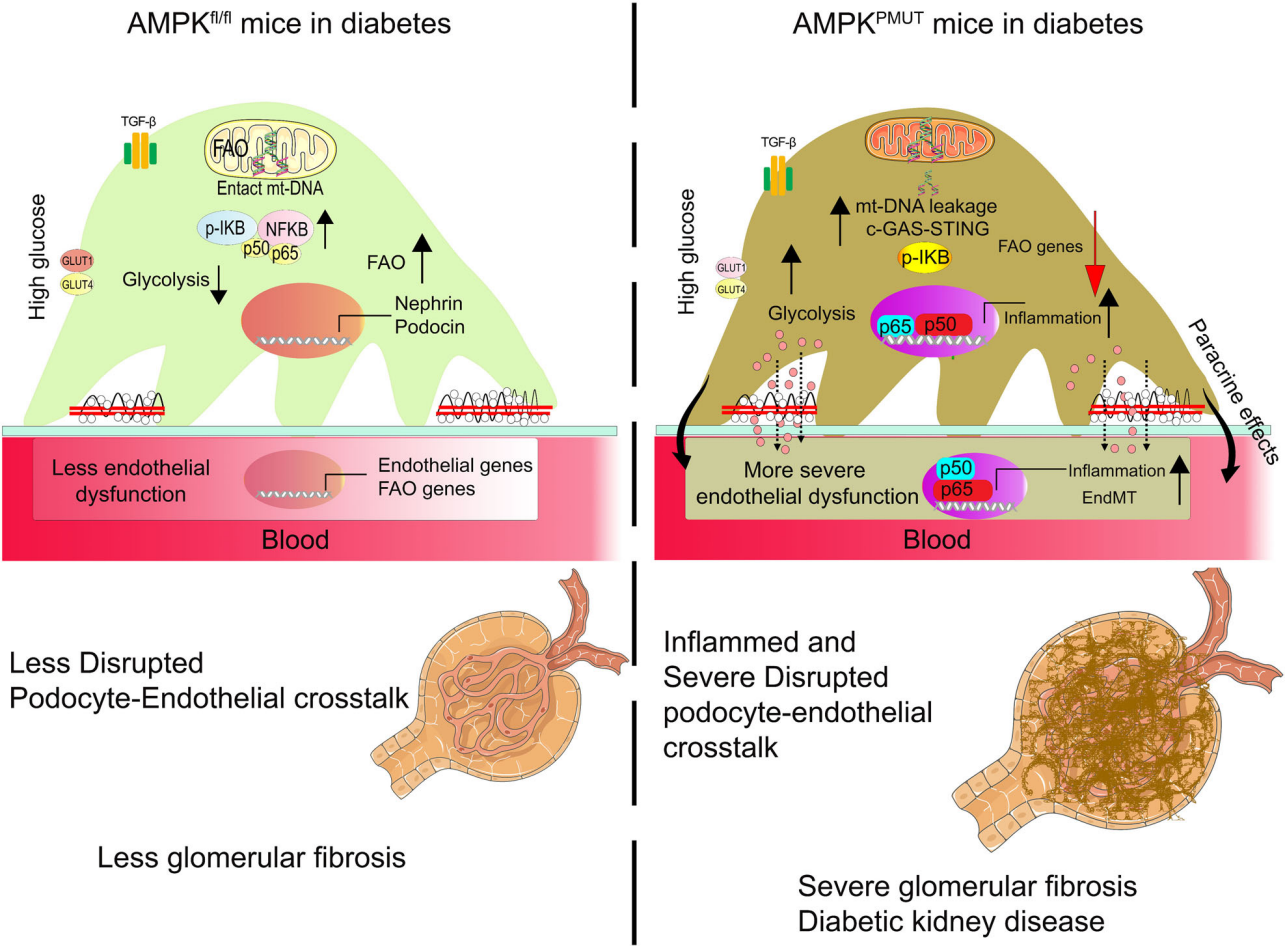
A hypothetical model of the proposed AMPK function in type 1 diabetic glomerular podocytes. While AMPK is dispensable in maintaining podocyte and glomerular function under normal fed conditions, with type 1 diabetes, AMPK may play an important role in mitigating the progression of DKD. Loss of AMPK in podocytes exacerbated lipid deposition in diabetic glomeruli, which, in turn, damaged mitochondria, leading to mt-DNA leakage into the cytoplasm and activation of the cGAS-STING pathway. Activation of the STING pathway leads to p65 activation and enhances the transcription of genes associated with inflammation. Simultaneously, the loss of AMPK enhances aberrant glycolysis in the podocytes. These metabolic shifts in podocytes also drive EndMT, and their cumulative effects promote the progression of glomerular fibrosis under diabetic conditions.

As outlined in Fig. 9, while AMPK in podocytes is dispensable for physiological podocyte function under normal ad-lib-fed conditions, it plays an important role in maintaining the function of both podocytes and endothelial cells by reducing lipid toxicity and maintaining appropriate glucose levels, thereby mitigating the induction of aberrant metabolism under diabetes. In the absence of AMPK in podocytes, pathological suppression of FAO and aberrant glycolysis are further exacerbated in type 1 diabetes, leading to excessive lipid accumulation in podocytes and neighboring endothelial cells, mitochondrial damage, activation of the c-GAS-STING pathway, and inflammation. These cumulative effects of metabolic shift and inflammation may drive EndMT in glomerular endothelial cells. AMPK has dozens of substrates that directly or indirectly regulate lipid and glucose homeostasis and inflammation, including hormone-sensitive lipase (HSL), adipose triglyceride lipase (ATGL), Sirtuin 1, Peroxisome Proliferator-Activated Receptor Gamma Coactivator 1α (PGC-1α), and p53, transcription factors [27, 108, 109]. It remains unclear which reduced downstream AMPK effector functions contribute to deteriorative effects on the podocytes and neighboring endothelial cells in pmut diabetic kidneys. However, the study supports the idea that AMPK activation would be a reliable approach to protect glomerular dysfunction from diabetes by maintaining better metabolism in podocytes as well as other neighboring glomerular cells, such as endothelial cells.

## Material and method

### Antibodies and reagents

Rabbit anti-pS6 (Cat: 5364), rabbit CPT1a (Cat: 12252), rabbit LC3A (Cat: 4599), and rabbit PKM2 (Cat: 4053) were purchased from Cell Signaling Technology. Mouse anti-WT1 (Cat: 05-753) was purchased from EMD. Rabbit anti-p-ATG1/ULK-1 (Cat: 80218-1-RR), rabbit anti-PFKFB3 (Cat: 13763-1-AP), rabbit anti-Fibronectin (Cat: 15613-1-AP), and anti-p-IKB (Cat: 82349-1-RR) were purchased from Proteintech. Goat anti-CD31 (Cat. AF3628) was purchased from R&D Systems. Rabbit anti-TFAM (Cat: PA5-29571), rabbit anti-Vimentin (Cat: MA5-35320), and rabbit anti-Collagen I (Cat: PA5-95137) were obtained from Invitrogen. Fluorescence-, Alexa Fluor 647–, and rhodamine-conjugated secondary antibodies were obtained from Life Technologies. Streptozotocin was purchased from Sigma.

### Animal experiments

For the podocyte-specific loss of function of AMPK, we bred the *Ampk α1* ^*fl/fl*^*α2* ^*fl/fl*^ mice with *podocin-Cre* mice to generate mice with deletion of all catalytic subunits of AMPK, specifically in podocytes (pmut mice). Previously, *Ampk α1* ^*fl/fl*^*α2* ^*fl/fl*^ mice were generated by the Morrison group [110]. All mice were on the C57BL/6 background. We analyzed both males and females in the experiments on aged mice. Given the worsened diabetic kidney disease phenotypes observed in male compared with female mice, male mice were used in the diabetic mouse experiments. The mice were housed in polypropylene cages, with three-to-four mice per cage at the animal facility of the Life Sciences Institute, University of Michigan. All animal experiments were approved by the Institutional Animal Care and Use Committee (IACUC) at the University of Michigan (#PRO00011945) and Kanazawa Medical University animal protocols (#2014-89, #2013-114, and #2014-101).

#### STZ diabetic mice

Type 1 diabetes was induced in 10-week-old *Ampk α1* ^*fl/fl*^*α2* ^*fl/fl*^, *podocin-Cre* (pmut) mice and control littermates, *Ampk α1* ^*fl/fl*^*α2* ^*fl/fl*^ (WT) mice, with five consecutive intraperitoneal doses of Streptozotocin (STZ: 50 mg/kg in 10 mmol/L citrate buffer (pH 4.5)), and the mice were monitored over 24 weeks [111–114]. Urine samples for albumin and creatinine levels were collected using metabolic cages at the indicated points (8, 12, and 20 weeks after STZ treatment). Before sacrifice, mice were weighed, and blood glucose levels were measured using glucose strips.

#### HFD + STZ diabetic mice

10-week-old mice (WT and pmut) were fed a high-fat diet for 8 weeks and then treated with three consecutive low-dose STZ (35 mg/kg). After another seven weeks of HFD, an IPGTT (1 g/kg glucose) was performed, and 24-hour urine was collected to determine ACRs.

#### Metformin treatment

Type 1 diabetes was induced by STZ in 8-week-old mice. At 12 weeks old, the diabetic mice were treated with or without metformin (100 mg/kg) for 4 weeks.

In preliminary studies, we detected a significant difference in the severity of glomerular fibrosis, our primary outcome, with 5 animals/group [63, 107, 115]. Therefore, we used that minimum sample size (*n* > 5) for all in vivo experiments. After the STZ injection, blood glucose (BG) levels were analyzed using glucose strips at 7 days, and non-responders (non-diabetic mice with BG < 200 mg/dl) were excluded from the experiment.

### In vivo physiologic studies

Urinary albumin and creatinine concentrations were determined using a mouse-specific Albuwell M and Creatinine Companion kit (Exocell). Glomerular tuft area, PAS-positive mesangial area, and other pixel densities obtained by immunohistochemical experiments were measured using ImageJ software.

### Isolation of glomeruli

The podocyte isolation was performed as previously described [116]. Adult C57BL/6 mice at 8-14 weeks of age were used in this study. For glomerular isolation with the differential adhesion method, a mouse was euthanized by an overdose of Isoflurane. The two kidneys from one mouse were harvested, and the medulla was removed carefully. The renal cortices were minced into tiny particles by chopping with a razor blade in 0.5 ml of HBSS and then digested with collagenase type V at 37 °C for 15–20 min, with pipetting at 5-minute intervals. Digestion was stopped by adding 4 mL of DMEM supplemented with 10% FBS. The digested tissue was spun down at 290 g for 1 min at 4 °C and resuspended in 5 ml of HBSS. The resulting mixture was transferred onto a pre-wetted 100 µM plastic cell strainer. The strainer was washed with 5 ml of fresh cold HBSS. All filtrate was collected and washed through a pre-wetted 75 µM plastic cell strainer with 35 ml of fresh cold HBSS. The filtrate (50 ml) was collected and rewashed through another prewetted 40 µM plastic cell strainer with the fresh cold HBSS. The retained glomerular and tubular fragments on the top of the 40 µM strainer were rinsed into a clean cell culture dish. After 12 min of settling, large, fragmented tubules adhered to the bottom of the dish, leaving most glomeruli and small, fragmented tubules floating in the supernatant. By gently swirling the culture dish, the glomeruli-enriched supernatant was collected and transferred with a plastic Pasteur pipette onto a new pre-wetted 40 µM strainer. After a wash with 25 ml of fresh cold HBSS to remove small tubular fragments, the retained glomeruli on the top of the 40 µM strainer were rinsed into another clean cell culture dish for a second adhesion to remove residual large tubular fragments. The supernatant containing highly purified glomeruli was collected and centrifuged at 290 × *g* for 5 min at 4 °C in a swing-out rotor centrifuge.

### Kidney histology

Sirius red, periodic acid-Schiff, and Masson-Trichrome staining were performed by the University of Michigan Pathology Core, and the slides were visualized using an Aperio imaging system. Masson-Trichrome-stained sections were evaluated using ImageJ software, and the fibrotic areas were estimated. For Sirius red staining, deparaffinized sections were incubated with picrosirius red solution for 1 hour at room temperature. The slides were washed twice with acetic acid solution for 30 s per wash. Then, the slides were dehydrated in absolute alcohol three times. The slides were cleared in xylene and mounted with a synthetic resin. For each mouse, images of eight different fields of view were evaluated at ×40 magnification, and 15 to 20 stained glomeruli from each mouse were analyzed. Masson-Trichrome stain, a relative area of fibrosis, and Sirius red relative collagen deposition were analyzed to calculate glomerular fibrosis and glomerular collagen deposition, respectively. Glomerular surface area was calculated using traced glomeruli and ImageJ algorithms.

### Immunofluorescence

Paraffin-embedded kidney sections (5 μm) were used for immunofluorescence staining; double-positive labeling with pS6, LC3, Vimentin, Desmin, Collagen I, Biodipy, CPT1a, PFKFB3, PKM2, TFAM, pIκB, p65, or pATG1 with WT1 for podocytes; and Vimentin, Fibronectin, CPT1a, PFKFB3, or PKM2 and CD31 for endothelial cells was performed.

### Image analysis and quantification

Data were analyzed using ImageJ (Fiji, version 1.54p). Immunofluorescence intensity was determined by ImageJ by splitting the color channel, and the intensity of the signal was divided by glomerular area (intensity/µm^2^). The number of LC3 puncta in the glomerulus was determined by Fiji Image J. Briefly, glomerular regions of the fluorescence images were cropped, their color channels were split, and the background was reduced. The ratio (the number of LC3 puncta/glomerular area) was automatically determined using H-watershed (hmin=7.0, thresh=18, peakflooding=35, allowsplitting=true) and monitored the number of particles (size=0-50: less than 8 µm^2^).

### Oil red O staining

Briefly, 5 μm kidney sections were dried at room temperature for 15 min, then fixed in prechilled acetone for 10 min. Slides were washed three times with PBS for 5 min and lastly rinsed in 60% isopropanol for 5 min. Lipids were stained by incubating slides in fresh Oil Red O working solution for 60 min at room temperature, followed by rinsing in 60% isopropanol for 5 s. Slides were washed three times with PBS and for 5 min with water, then counterstained with hematoxylin. Last, slides were washed in 70% ethanol, mounted with mounting media, and immediately imaged.

### EndMT detection

Paraffin-embedded kidney sections (5 μm) were used to detect EndMT. Cells undergoing EndMT were detected by double-positive labeling for CD31 and Vimentin and CD31 and Fibronectin. Sections were analyzed and quantified by fluorescence microscopy.

### Transmission electron microscopy

The sample preparations and electron microscopic analyses were performed at the Biomedical Research Core at the University of Michigan. For sample preparation, mice were anesthetized and perfused with Sorensen phosphate buffer containing 4% PFA and 2.5% glutaraldehyde, and the kidneys were isolated. The processed samples were analyzed using field-emission scanning electron microscopes. ImageJ was used to quantitatively analyze electron micrographs. Foot processes were measured from at least 50 μm of GBM for each mouse. Podocyte foot processes and GBM thickness were analyzed by ImageJ. Images were blinded by assigning integer numbers before evaluation by someone other than the scorer.

### Cell treatment and immunoblotting

Mouse temperature-sensitive podocytes (kindly gifted by Dr. Peter Mundel) were differentiated at 37 °C. Contamination with Mycoplasma was routinely checked using the e-Myco^TM^ Mycoplasma PCR Detection kit V2.0 (Boca Scientific, Cat# 25235). The cells were treated with MK8722 (2.5 μM), an AMPK activator, under low-glucose (5.5 mM) or high-glucose (25 mM) culture conditions for 48 hours. Cells were lysed in NP-40 cell lysis buffer (1% NP-40, 40 mM HEPES pH 7.5, 120 mM NaCl, 50 mM NaF, 10 mM β-glycerophosphate, 10 mM sodium pyrophosphate, 1 mM EDTA, 1× Protease Inhibitor Cocktail (Roche, Cat.NO. 11873580001)). Lysates were then boiled in SDS sample buffer (20 mM Tris, pH 6.8, 2% SDS, 0.01% bromophenol blue, 10% glycerol, 5% 2-mercaptoethanol) and subjected to SDS-PAGE and immunoblotting with the indicated antibodies.

### mRNA isolation and quantitative polymerase chain reaction

Total RNA was isolated using the standard Trizol protocol. RNA was reverse transcribed using the iScript cDNA Synthesis kit (Bio-Rad), and quantitative polymerase chain reaction was performed on a Bio-Rad C1000 Touch thermal cycler using the resultant cDNA, quantitative polymerase chain reaction master mix, and gene-specific primers. The primers used are given in Table S1. Gene expression was normalized to the housekeeping gene actin and is presented as a fold change. All primers were synthesized by IDT.

### Statistical analysis

All values are expressed as mean ± SEM and analyzed using GraphPad Prism 8 (GraphPad Software Inc., La Jolla, CA). The significance of differences was determined using an unpaired two-tailed Student’s *t* test when comparing two independent groups. The one-way ANOVA, followed by Tukey’s test, was employed to analyze the significance when comparing multiple independent groups. The post-hoc tests were run only if F achieved *p* < 0.05 and there was no significant variance inhomogeneity. The declared group size is the number of independent values, and the statistical analysis was performed using these independent values. In each experiment, *N* represents the number of separate experiments (in vitro) and the number of mice (in vivo). Technical replicates were used to ensure the reliability of single values. Randomization was not performed in this study. In our experimental design, we ensured that equal or sufficient numbers of mice from each group were treated with each therapy that we tested. Data analyses were blinded. All p-values were adjusted for multiple comparisons using the Bonferroni correction, and the adjusted values were reported. p values of less than 0.05 were considered statistically significant: *p < 0.05, **p < 0.01, ***p < 0.001, ****p < 0.0001

## Supplementary information

Online Supplemental File

Original Data

## Data Availability

The datasets used and/or analyzed during the current study are available from the corresponding author (Swayam Prakash Srivastava; spsr@umich.edu) upon request.
