# Supplementary material for: AMPK is dispensable for physiological podocyte and glomerular functions but prevents glomerular fibrosis in experimental diabetes
Source: Cell Death Discov. 2026 Mar 28;12:204. doi: 10.1038/s41420-026-03078-y (PMC13153347; doi:10.1038/s41420-026-03078-y)
Supplement: Supplementary file 1 — Online Supplemental File [file 41420_2026_3078_MOESM1_ESM.pdf]

Online Supplemental File

AMPK is dispensable for physiological podocyte and glomerular functions but prevents glomerular fibrosis in experimental diabetes

Swayam Prakash Srivastava[1#], Olivia Kopasz-Gemmen[1], Abhiram Kunamneni[1], Aaron Thurman[1], Shota Yoshida[1], Eden Ozkan[1], Vinamra Swaroop[1], Rahul Nanwani[1], Ajan Arora[1], Arya Joshi[1], Om Khuperkar[1], Mariam Hamed[1], Mihir Suresh Bharadwaj[1], Niloy Islam[1], Adesh Urval[1], Junying Wang[1], Sungki Hong[1], Keizo Kanasaki[2], Daisuke Koya[2], Ken Inoki[1,3,4,#]

1. Life Sciences Institute, University of Michigan, 210 Washtenaw Avenue, Ann Arbor, Michigan, 48109-2216, U.S.A.
2. Department of Diabetology and Endocrinology, Kanazawa Medical University, Uchinada 920-0293, Japan.
3. Department of Molecular and Integrative Physiology, University of Michigan Medical School, 1137 E. Catherine St., Ann Arbor, Michigan, 48109-5622, U.S.A.
4. Department of Internal Medicine, University of Michigan Medical School, 1500 East Medical Center Drive, Ann Arbor, Michigan, 48109-5368, U.S.A.

# Corresponding authors: spsr-at-umich.edu and inokik-at-umich.edu

[#]-Correspondence:

Swayam Prakash Srivastava, PhD

Life Sciences Institute, University of Michigan

Email: spsr@umich.edu; swayam.cdri@gmail.com

&

Ken Inoki, MD, PhD

Life Sciences Institute, University of Michigan

Email: inokik@umich.edu

Supplemental Figure 1.

S1A. Quantitative qPCR analyses of *Ampk* $\alpha 1$ and *Ampk* $\alpha 2$ transcripts in the indicated glomeruli from wild-type (WT) and pmut (PMUT) mice are shown. Data are shown as mean±SEM. ***p<0.001, N=5.

S1B. Double immunofluorescence staining of pULK1/ATG1 (S556) and WT1 was shown. While recent studies argue for the role of phosphorylation of S566 in ATG1 (ULK1) in regulating ATG1 activity, AMPK is a key kinase that phosphorylates S556. Representative images are shown.

S1C. Blood glucose level was determined in 2-year-old WT and pmut mice. Data are shown as mean±SEM. N=8. ns indicates no significance.

S1D. Oil O Red staining was performed on kidney tissues from 2-year-old WT and pmut mice. Representative images are shown.

Supplemental Figure 2.

S2A and S2B. Double immunofluorescence staining of M2-type macrophages (CD206), M1-type macrophages (iNOS), with WT1 was shown. ITC-labelled WT1; Rhodamine-labelled CD206 and iNOS; and DAPI-blue nucleus. Representative images are shown. The scale bar is 50 µm (S2A). The intensity of the signals within the indicated glomeruli was quantified. Data are shown as mean±SEM. *p <0.05, N=6 (S2B).

Supplemental Figure 3.

S3A. Schematic chart demonstrating the treatment protocol for the AMPK activator MK8722 (2.5 µM) in low-glucose- and high-glucose-cultured mouse podocyte cells. After 48h, cells were harvested for western blot analysis and qPCR analysis.

S3B. Western blot analysis of p-AMPK (pT172), AMPK, collagen I, and fibronectin in MK8722-treated low-glucose and high-glucose cultured mouse podocyte cells.

S3C and **S3D.** qPCR analysis of *Collagen I, Vimentin, TgfβR1* (S3C), *Il-1β, Il-6,* and *Nf-κb1* transcripts in the MK8722-treated low-glucose and high-glucose cultured mouse

podocyte cells. Data are shown as mean±SEM, ***p <0.001, ****p<0.0001, N=6. ns indicates no significance.

Supplemental Figure 4. Metformin treatment in diabetic mice.

S4A. A schematic diagram showing the experimental design analyzing the effect of metformin in control and STZ-induced diabetic mice.

S4B. Immunohistochemistry analysis of p-AMPK (pT172) in the kidneys of metformin-treated diabetic mice. N=6/group was analyzed. The glomerular intensity of p-AMPK signal was quantified. Data are shown as mean±SEM, **p <0.01.

S4C. Masson Trichrome (MTS) staining analysis in the kidneys of metformin-treated diabetic mice. Relative area fibrosis was measured using ImageJ software. N=6/group was analyzed. Data are shown as mean±SEM, ***p <0.001.

Supplemental Figure 1.

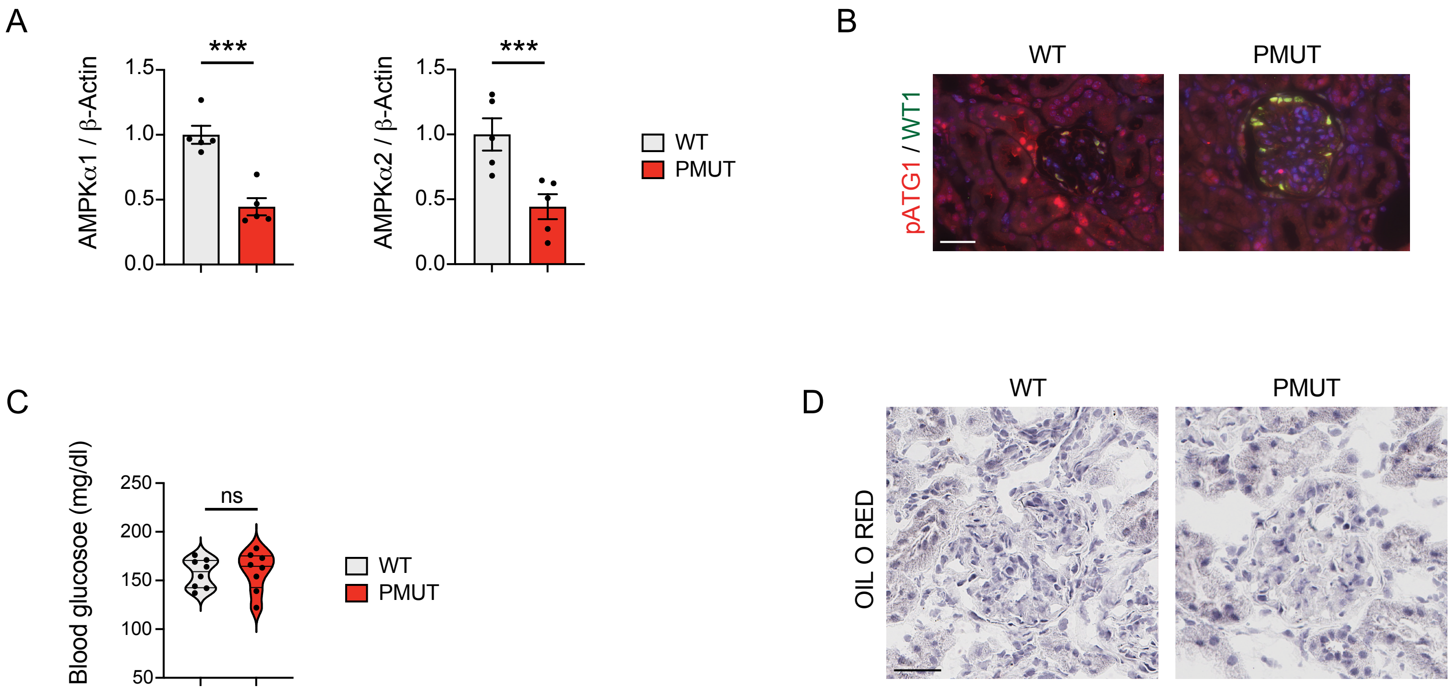

Supplemental Figure 2 .

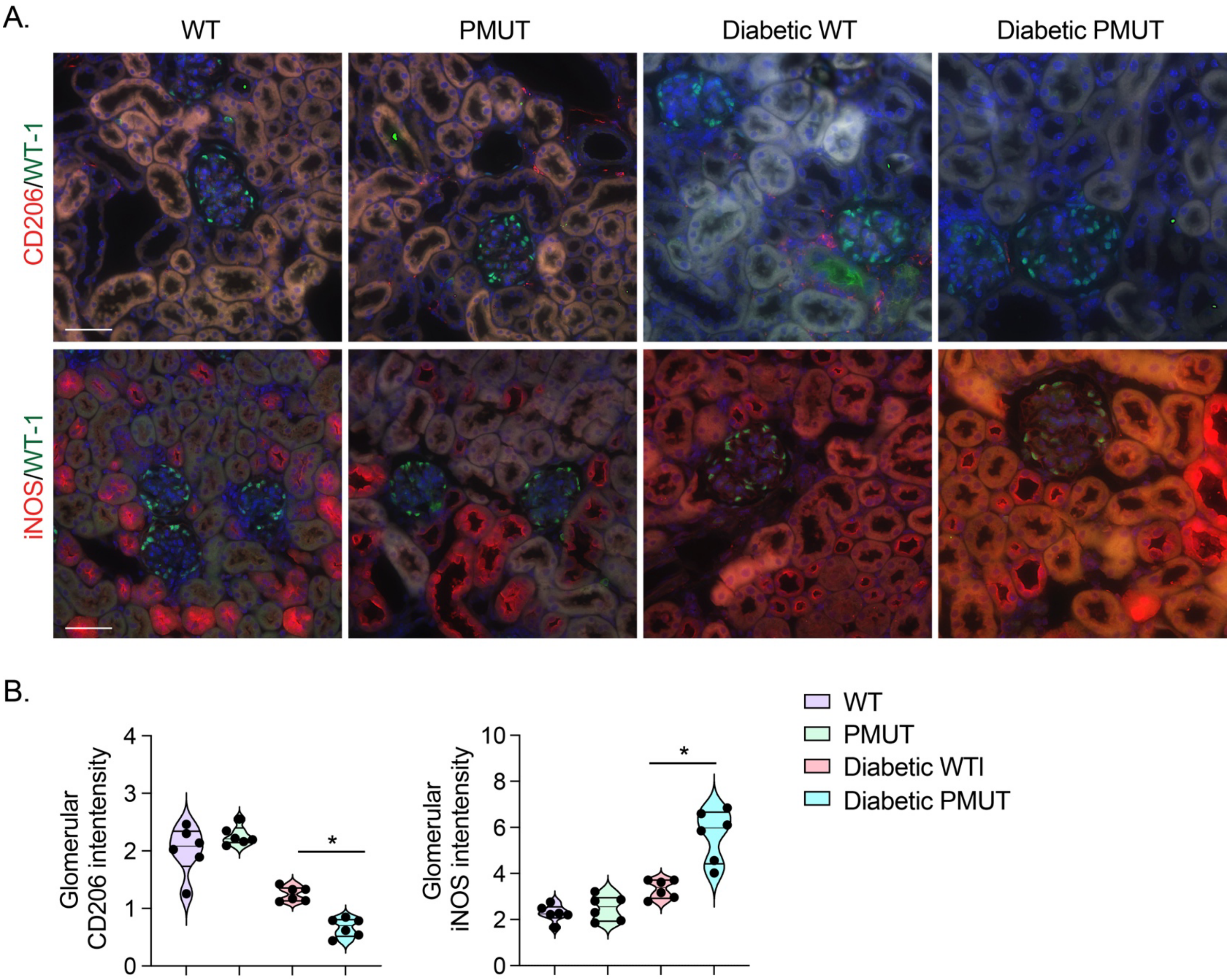

Supplemental Figure 3.

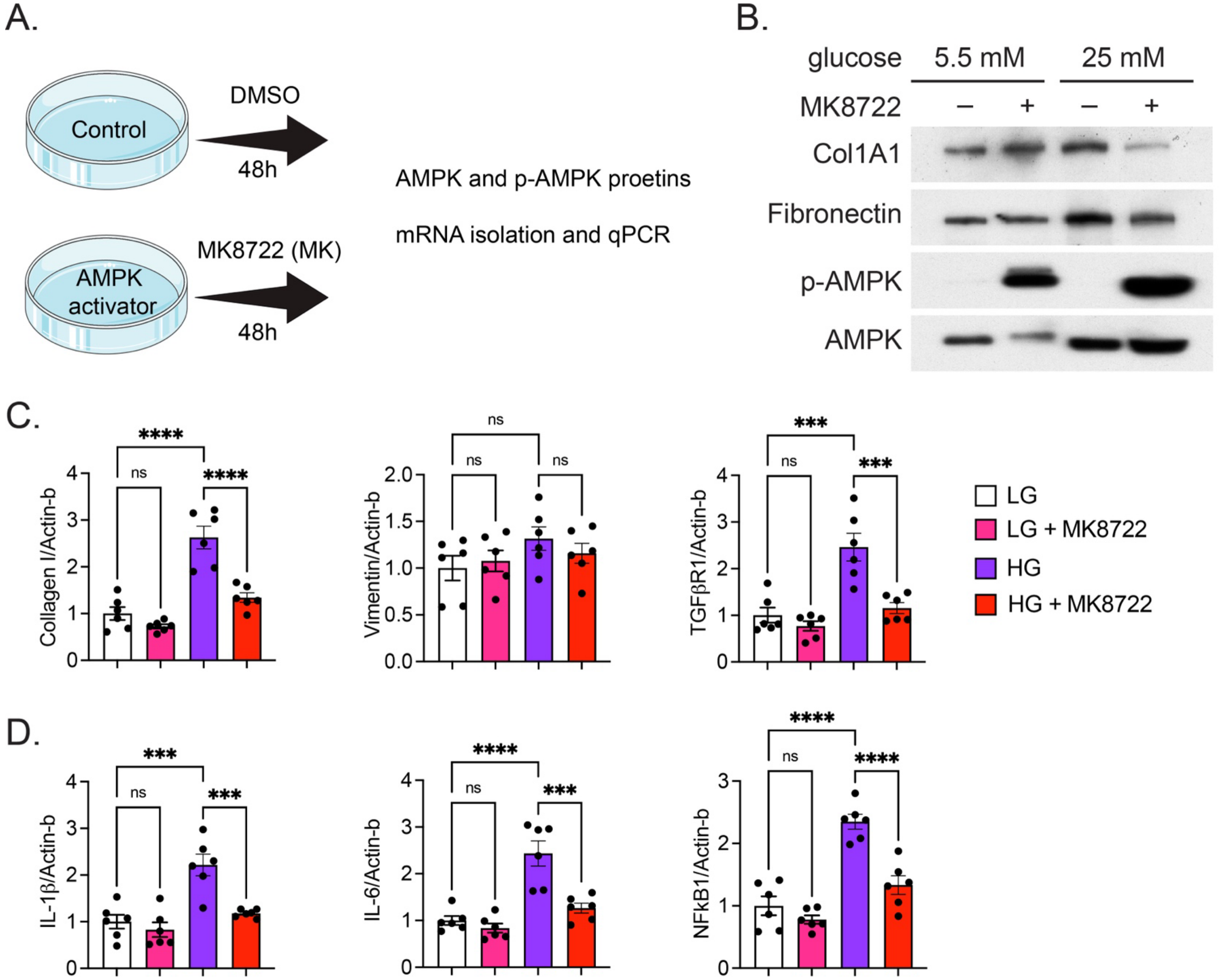

Supplemental Figure 4.

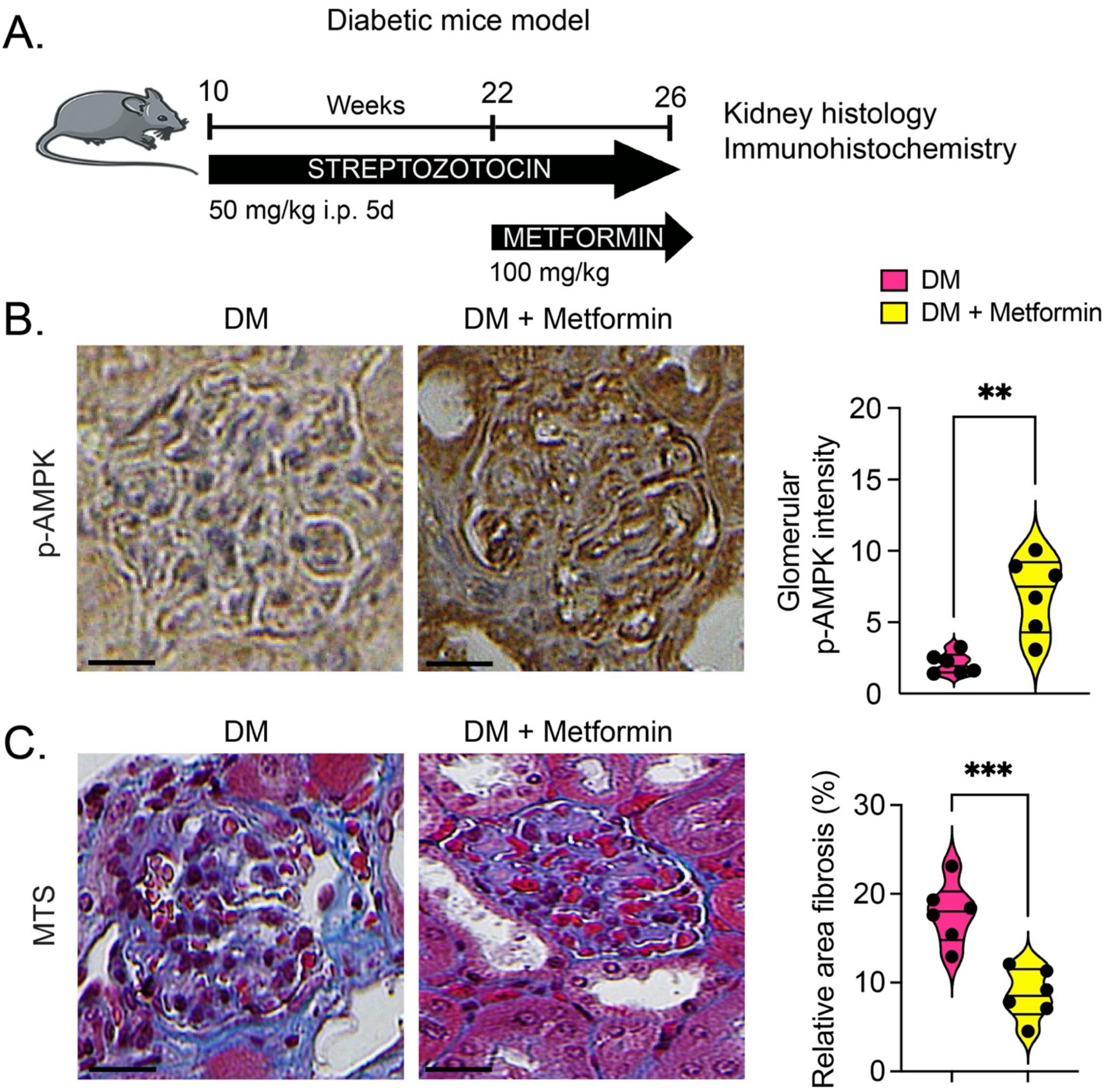

Table S1: Sequences mouse primers

Gene name	Forward primer	Reverse primer
NFkB1	5′-GCTGCCAAAGAAGGACACGACA	5′-GGCAGGCTATTGCTCATCACAG
IL-1β	5′-TGGACCTTCCAGGATGAGGACA	5′-GTTCATCTCGGAGCCTGTAGTG
IL-6	5′-TCTGAAGGACTCTGGCTTTG	5′-GATGGATGCTACCAAACTGGA
Ndufv2	5’-TGGATGGCTACCTATCTCCGCT	5’-GGTACTTCCCAACTGGCTTTCG
PDHA1	5′-GTGAGAACAACCGCTATGGCATG	5′-CGCAAACTTTGTTGCCTCTCGG
LDH	5’-ACGCAGACAAGGAGCAGTGGAA	5’-ATGCTCTCAGCCAAGTCTGCCA
mt-Co1	5’-GCCCCAGATATAGCATTCCC	5’-GTTCATCCTGTTCCTGCTCC
mt-Cyb	5’-AGTAGACAAAGCCACCTTGA	5’-CCGCGATAATAAATGGTAAG
TFAM	5’-GAGCAGCTAACTCCAAGTCAG	5’-GAGCCGAATCATCCTTTGCCT
TMEM173	5’-TTTGCCATGTCACAGGATGC	5’-ATGAGGCGGCAGTTATTTCG
Mb21d1	5’-TGGTGGGAAGAGTGGTGATTTC	5’-TGCATTCCAATGGCAGAAGC
β-actin	5’-AGAAGCTGTGCTATGTTGCTCTA	5’-ACAGGATTCCATACCCAAGAAGGA
