# Supplementary figures and images for: AMPK is dispensable for physiological podocyte and glomerular functions but prevents glomerular fibrosis in experimental diabetes
Source: Cell Death Discov. 2026 Mar 28;12:204. doi: 10.1038/s41420-026-03078-y (PMC13153347; doi:10.1038/s41420-026-03078-y)

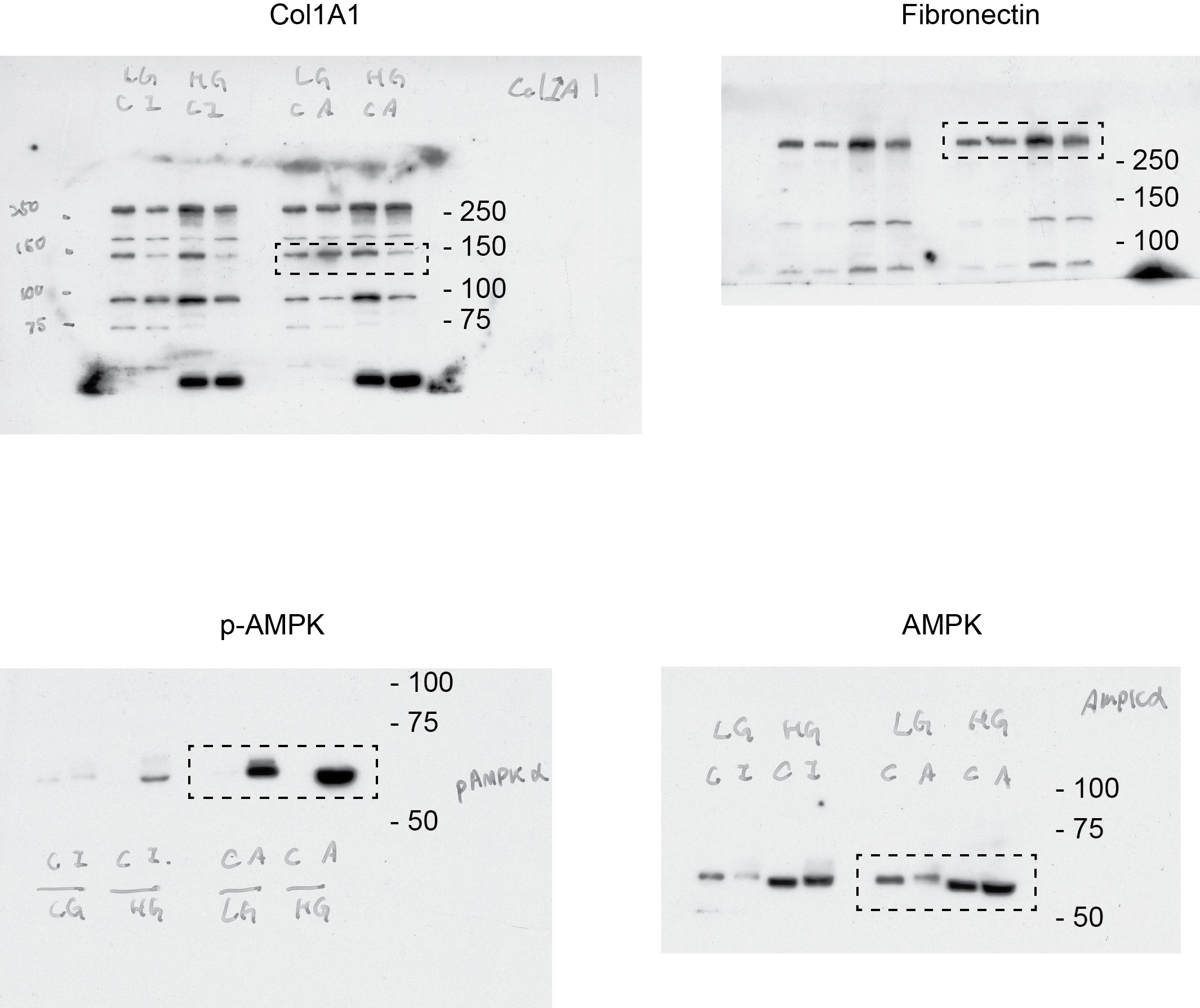

Supplement: Supplementary file 2 — Original Data [file 41420_2026_3078_MOESM2_ESM.jpg]
